# Deep learning detection of nanoparticles and multiple object tracking of their dynamic evolution during in situ ETEM studies

**DOI:** 10.1038/s41598-022-06308-2

**Published:** 2022-02-15

**Authors:** Khuram Faraz, Thomas Grenier, Christophe Ducottet, Thierry Epicier

**Affiliations:** 1 Université de Lyon, UJM-Saint-Etienne, CNRS, Institut d’Optique Graduate School, Laboratoire Hubert Curien UMR 5516, 42023 Saint-Etienne, France; 2 Université Lyon, INSA-Lyon, Université Claude Bernard Lyon 1, CNRS, MATEIS, UMR 5510, 69621 Villeurbanne Cedex, France; 3 Université Lyon, INSA-Lyon, Université Claude Bernard Lyon 1, CNRS, CREATIS, UMR 5220, INSERM U1206, 69621 Villeurbanne Cedex, France; 4 Université Lyon, Université Claude Bernard Lyon 1, CNRS, IRCELYON, UMR 5526, 69626 Villeurbanne, France

**Keywords:** Materials for energy and catalysis, Imaging techniques, Scientific data, Transmission electron microscopy, Characterization and analytical techniques, Heterogeneous catalysis

## Abstract

In situ transmission electron microscopy (TEM) studies of dynamic events produce large quantities of data especially under the form of images. In the important case of heterogeneous catalysis, environmental TEM (ETEM) under gas and temperature allows to follow a large population of supported nanoparticles (NPs) evolving under reactive conditions. Interpreting properly large image sequences gives precious information on the catalytic properties of the active phase by identifying causes for its deactivation. To perform a quantitative, objective and robust treatment, we propose an automatic procedure to track nanoparticles observed in Scanning ETEM (STEM in ETEM). Our approach involves deep learning and computer vision developments in multiple object tracking. At first, a registration step corrects the image displacements and misalignment inherent to the in situ acquisition. Then, a deep learning approach detects the nanoparticles on all frames of video sequences. Finally, an iterative tracking algorithm reconstructs their trajectories. This treatment allows to deduce quantitative and statistical features about their evolution or motion, such as a Brownian behavior and merging or crossing events. We treat the case of in situ calcination of palladium (oxide) / delta-alumina, where the present approach allows a discussion of operating processes such as Ostwald ripening or NP aggregative coalescence.

## Introduction

### Context of the study

In many fields of nanoparticle (NP) research, one key parameter allowing the understanding of elementary mechanisms, then the optimization of the processes being studied, consists in obtaining a quantitative metrological analysis of the population of NPs. Static parameters, like size, shape, 2D or 3D distribution are the first type of features that are usually determined^1,2^, using a wide variety of experimental techniques that give access to the adequate spatial resolution, such as light scattering^3,4^, Atomic Force Microscopy (AFM)^5,6^, Small Angle X-Ray Scattering (SAXS)^7^, Scanning and Transmission Electron Microscopy (SEM^8–11^ and TEM^12–17^) and EM approaches combined to other techniques^18–20^. When dealing with NPs of a few nanometers, Transmission Electron Microscopy (TEM) is one of the best characterization tools. The recent development of so-called Environmental TEM (ETEM), where the object is no longer studied under high vacuum as in a conventional TEM, but under gas^21^ or liquid^22^ and possibly in temperature, has made this technique become one of the most powerful Swiss army knives for in situ characterization of the dynamic evolution of NPs in gas (e.g.^23,24^) or in liquids (e.g.^25–28^). A typical application field is heterogeneous catalysis, where a population of controlled NPs is distributed on a solid support in order to catalyze specific gas or liquid chemical reactions^29,30^. In this context, it is of the outermost importance to provide quantitative data about the previously mentioned morphological parameters, but also on their dynamic evolution during in situ heat treatments, as required for conditioning the nanocatalysts, or, in fine, during the catalytic reaction itself as they can be performed in the ETEM^31–34^. One major difficulty to achieve such goals is to be able to analyze in a robust way a large number of objects in order to draw out conclusions with a sufficient statistical meaning. A basic and simple approach is to count and measure manually or semi-automatically a few particles during, typically, calcination or reduction treatments in temperature: this has been performed in some cases to study the growth of NPs during coalescence and/or Ostwald ripening ETEM^35–38^. Such a manual processing quickly becomes impractical when dealing with a large population in a dynamic approach in order to track realistically the evolution of the catalysts in time using video recording. The purpose of the present work is to develop algorithms and methodologies in order to treat automatically sequences of images recorded during in situ ETEM observations of metal-based nanocatalysts supported on an oxide media under gas at high temperatures. We first survey briefly the main algorithmic approaches employed so far.

### A brief survey of algorithmic approaches

The temporal tracking of moving objects along an image sequence is a well-known computer vision problem referred to as video tracking. This problem occurs in many applications from very different fields, e.g. human-computer interaction, security and surveillance, live-cell imaging, electron microscopy, etc. Best performing approaches rely on the tracking by detection paradigm: first, object positions are determined by running a specific detector over all the image frames. Second, data association is performed to link detections in the temporal dimension and construct all trajectories (or tracks). In the general context of microscopy, both steps are challenging and existing works generally focus on one particular part of the problem depending on the application. One specific feature for ETEM is that the high level of background noise makes the detection step difficult, which requires advanced methods to segment particles from the background. Recently, machine learning based methods and particularly deep learning based ones have been proposed as an accurate and efficient alternative to traditional methods^39,40^. Thus, particle detection and segmentation can be addressed using so-called semantic segmentation, which refers to the task of classifying all pixels of an image in a predefined semantic class (typically object or non-object for binary classification)^41^. Deep learning based methods allow to determine, using only the training data and its known ground truth, all the parameters of the network which minimizes a loss related to the task at hand^41^. Quite naturally, the popular semantic segmentation architecture U-Net^42^ has been proposed in various contexts^43^ as liquid-phase TEM^44^ or high-resolution TEM^45^. An important bottleneck of machine learning semantic segmentation is the requirement to have a huge set of reference images, annotated at the pixel level, to train the network. To solve this issue, few works so far have proposed to use simulated images for NPs detection or segmentation in TEM^44^ or SEM^46^. However, none of these works has accurately considered an uneven background. In this paper, we propose to use U-Net architecture with a specific training process based on simulated images of heterogeneous supported NPs, the support being either fully synthetic or obtained through real TEM images. Once the particles have been detected, a data association step must be performed to establish the correspondence between particles in successive frames. In a lot of works, the low density of particles and their relatively simple motion allow to solve this correspondence problem by a nearest neighbor principle^39^. However, in the case of a high density, heterogeneous motion, or when specific events, such as disappearance, merging or splitting of particles, can occur, the data association problem becomes very challenging. This situation has received particular attention in the field of live-cell imaging where time-lapse sequences are analyzed^47^. In this field, the problem is referred to as Single Particle Tracking (SPT)^48^. Generally, cell shape or motion can be integrated into the association process^49,50^ and best performing approaches rely on multiple-hypothesis tracking (MHT) as referred to in several reviews^51,52^. In this approach, the whole set of detections over the whole image sequence is considered. The tracking is then seen as an optimization problem aiming at selecting the most relevant set of tracks among all the possible solutions. As this problem is computationally intractable, some heuristics with lower computational cost have been introduced which provide suboptimal solutions. For example, Diatrack software^53^ tackles this complexity by globally and rapidly optimizing the matching of similar particles across multiple images through a graph structure^54^. However, the optimization is made only using a few successive frames. Finally, the work of Jaqaman et al.^55^ is among the few addressing complex situations with high particle density, particle motion heterogeneity, temporary particle disappearance, and particle merging and splitting. Their algorithm first links particles between consecutive frames and then links the resulting track segments into complete trajectories by solving a global combinatorial optimization problem. However, the two steps are independent and errors in the first step cannot be corrected in the second one. The approach adopted in the present work relies on a principle introduced in the context of video surveillance^56^ where the multi-target tracking problem, frequently referred to as Multiple Objects Tracking (MOT), is formulated as the minimization of a continuous energy. The energy function takes into account both physical constraints and the object’s appearance and behavior, and the optimization scheme alternates between continuous conjugate gradient descent and discrete trans-dimensional jump moves to explore the search space of varying dimensionality. The original algorithm, being devoted to tracking humans, does not take into account the peculiar constraints we have with NPs observed in ETEM (mass conservation, disappearance, merging and splitting, etc.) and we thus propose some important improvements of this method.

### Aim of the study

To summarize the above considerations, specifications of what should be a thorough analysis of NP dynamics during an in situ ETEM sequence can be listed as follows:

(i) perform a correct alignment of the image series without referring to the supported NPs in order to analyze properly their trajectories without having introduced biases by registration inaccuracies.
(ii) detect NPs on images from a dynamic series where the background might generally be non-uniform, as is the case of oxide supports for nanocatalysts, which severely impacts the signal-to-noise ratio. Most of existing quantitative studies via in situ (S)TEM concern rather homogeneous supports such as carbon or SiO$_2$ non-crystalline films^31,45,57^ or low density and poorly diffracting carbon particles^16,58^
(iii) Develop a robust and automatic method for tracking the motion of NPs in time and simultaneously detect and analyze events such as fusion (NPs agglomeration), disappearance or crossing of NPs (case of NPs on the upper and lower side of the support which may be superimposed in the projected views). To achieve this goal, measure the integrated intensity of the NPs in the case of incoherent, or almost incoherent, STEM imaging, since this value is related to the mass of the particles.

Some of these topics have been covered in previous works, for example (i)^12–17,45,57,58^ and (iii): Particle Tracking Analysis (PTA)^59^, mostly in the field of light microscopy^48,51^. Nevertheless, there is, to the best of our knowledge, no approach combining U-Net based detection and nearly optimal MHT accounting for all particularities of NPs in ETEM sequences. Furthermore, this original approach is pertinently applied in the practical case of the study of metallic nanocatalysts on oxide supports whereas numerous papers devoted to dynamics are focusing on the analysis of very few NPs (e.g.^24,26,27,35–38,59^) sometimes counted manually, which does not allow statistical features to be drawn out confidently. The present work intends to contribute to this objective. The calcination under oxygen of the system palladium (oxide) / delta-alumina (labeled Pd(O)@$\delta$-Al$_2$O$_3$ hereafter), used in various industrial catalytic reactions (see references in^38^), has been selected as a typical generic example of application.

## Results

### Numerical approach

#### Synopsis of our approach

**Figure 1:**
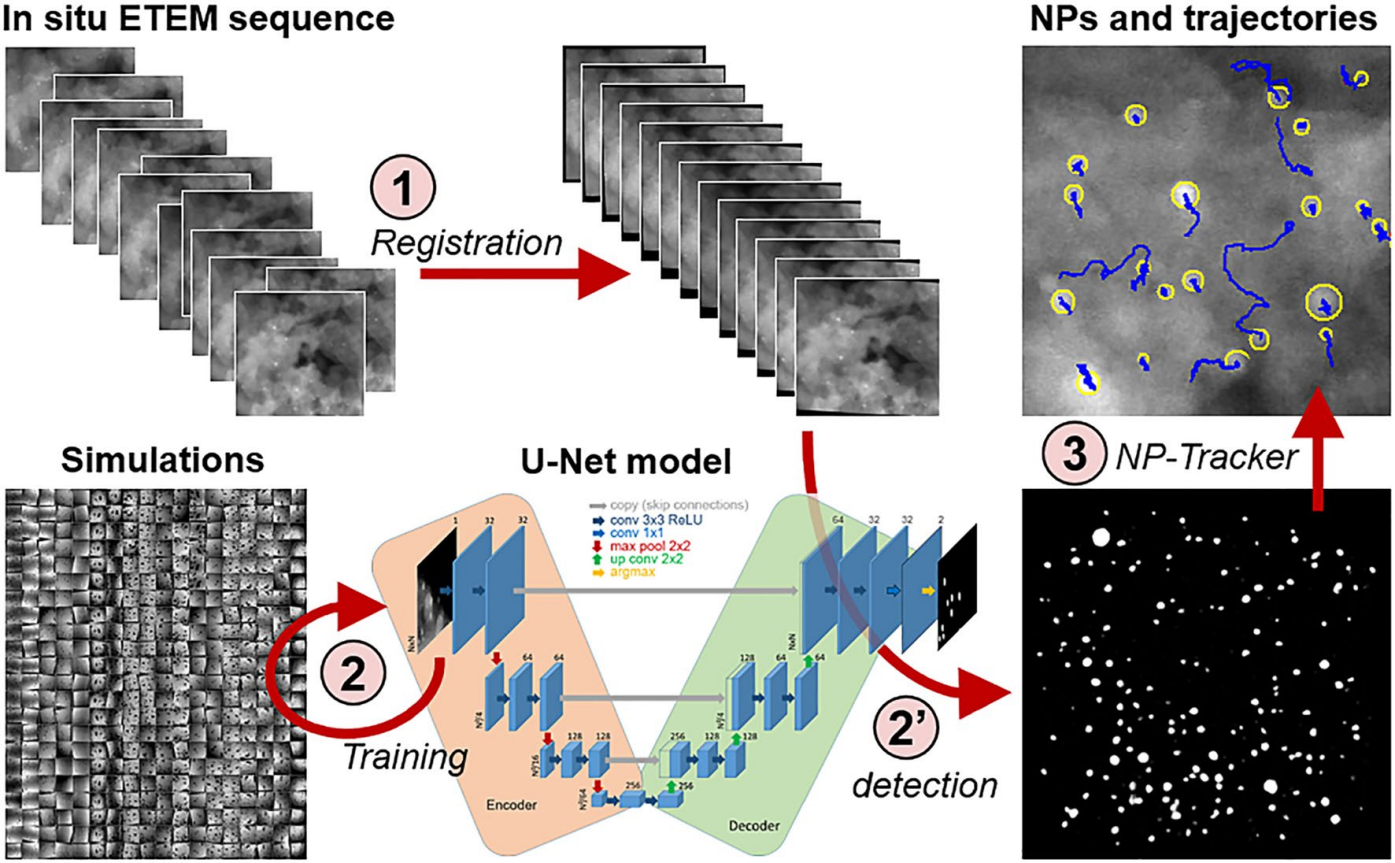
Synopsis of the algorithm. (1) Registration of the real input sequence, (2) training of a U-Net model with simulated images, (2’) detection and segmentation of particles in each frame using the model, (3) determination of trajectories using the dedicated NP-Tracker algorithm.

The first issue to solve is to register images of the sequence correctly. Due to sample drifts and possible intrinsic morphological evolutions during acquisition, the original images are not perfectly aligned along the sequence. Thus, a given fixed point of the scene can drift during the sequence, producing an apparent trajectory that can further be affected by scanning or local charging effects. These drifts must be perfectly corrected to retrieve the right trajectory of nanoparticles. We thus rely on an initial registration step to perform this alignment. Then the key elements of our approach is to combine a deep learning based detector to detect nanoparticles and a global optimization tracking method to find their trajectory. For the detector, we rely on the U-Net model, which is a fully convolutional deep neural network initially proposed by Ronneberger et al.^42^. After adequate training, this widely used semantic segmentation architecture is able to give an accurate segmentation of nanoparticles in spite of the high level of noise of our images. However, having an adequate dataset for training is not obvious because it requires having a large set of images together with a reference binary image (called the segmentation ground truth) indicating whether each pixel belongs to a particle or to the background. To create this dataset, we propose to generate realistic images simulating populations of spherical particles on a substrate together with their reference segmentation (directly available as we know the position and size of each particle). The second key component of our approach is a global optimization based tracking algorithm to determine the trajectory of each particle along the whole image sequence. We specifically adapt the Continuous Energy Minimization (CEM) tracker proposed by Milan et al.^56^ to account for the specific apparent behavior of imaged nanoparticles. Indeed, the nanoparticles can cross together in projection if they are respectively lying on the top and bottom surfaces of the supporting substrate. They can also grow by aggregation, merge with other particles or eventually shrink or even disappear due to diffusion into the support. Dedicated energy terms have then to be introduced in the algorithm to model these various effects. In summary, Fig. 1 presents the three steps of our pipeline: (1) the registration of the original sequence, (2) and (2’) the training of the U-Net model using simulated images and the inference step to detect NPs, (3) the CEM-based tracking, labeled NP-Tracker hereafter. The following subsections are presenting each of these steps more thoughtfully. Illustrations in Fig. 1 refer to an experimental sequence labeled ’Pd250’, which was acquired during an in situ calcination of a Pd(O)@$\delta$-Al$_2$O$_3$ sample under 2.2 mbar of oxygen at 250$^\circ$ C in the ETEM (this STEM image series is provided as supplementary video Video01.avi).

**Figure 2:**
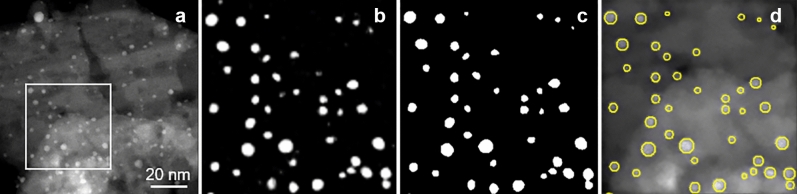
From U-Net segmentation to particle detection. (**a**) First frame of the Pd250 series; the field of view is about 130x130 $nm^{2}$). (**b**) U-Net prediction (probability map) for the enlarged white frame in (**a**). (**c**) Binary image. (**d**) Segmented particles represented by disks of an equal surface.

#### Deep learning U-Net based segmentation for nanoparticles detection

Automatic image segmentation, which is the delineation of objects in an image, has always been a complex but crucial task for many image analysis applications. Typically, a segmentation step is used here to locate and outline the nanoparticles present in the images. According to recent advances in machine learning, a deep learning approach is used here to perform the segmentation. We chose the U-Net network architecture which was originally proposed for semantic segmentation of the medical images^42^ and which is now derived in many ways^60^. We use a close to the original version of U-Net with 5 levels, 32 filters for the first level and 31,093,921 parameters, to segment automatically the nanoparticles (see architecture sketched in Fig. 1). Such a U-Net takes an image as input and produces a probability map of the presence of nanoparticles as output. After applying a threshold on this probability map, a post-processing step avoids close particles appearing as a single segmented object. To do so, we use a splitting algorithm based on the detection of the ultimate eroded points^61^. Figure 2 illustrates these different steps and the obtained result. This type of machine learning approach requires a distinction between two different phases: training and inference. The training phase will allow the network to gradually learn how to perform the task entrusted to it. In order to obtain a usable network, this phase requires a large dataset of images with their segmentation. The inference phase relies on a trained network and will thus produce results automatically on new data. The training strategy is explained in a specific sub-section hereafter.

#### Baseline image registration

**Figure 3:**
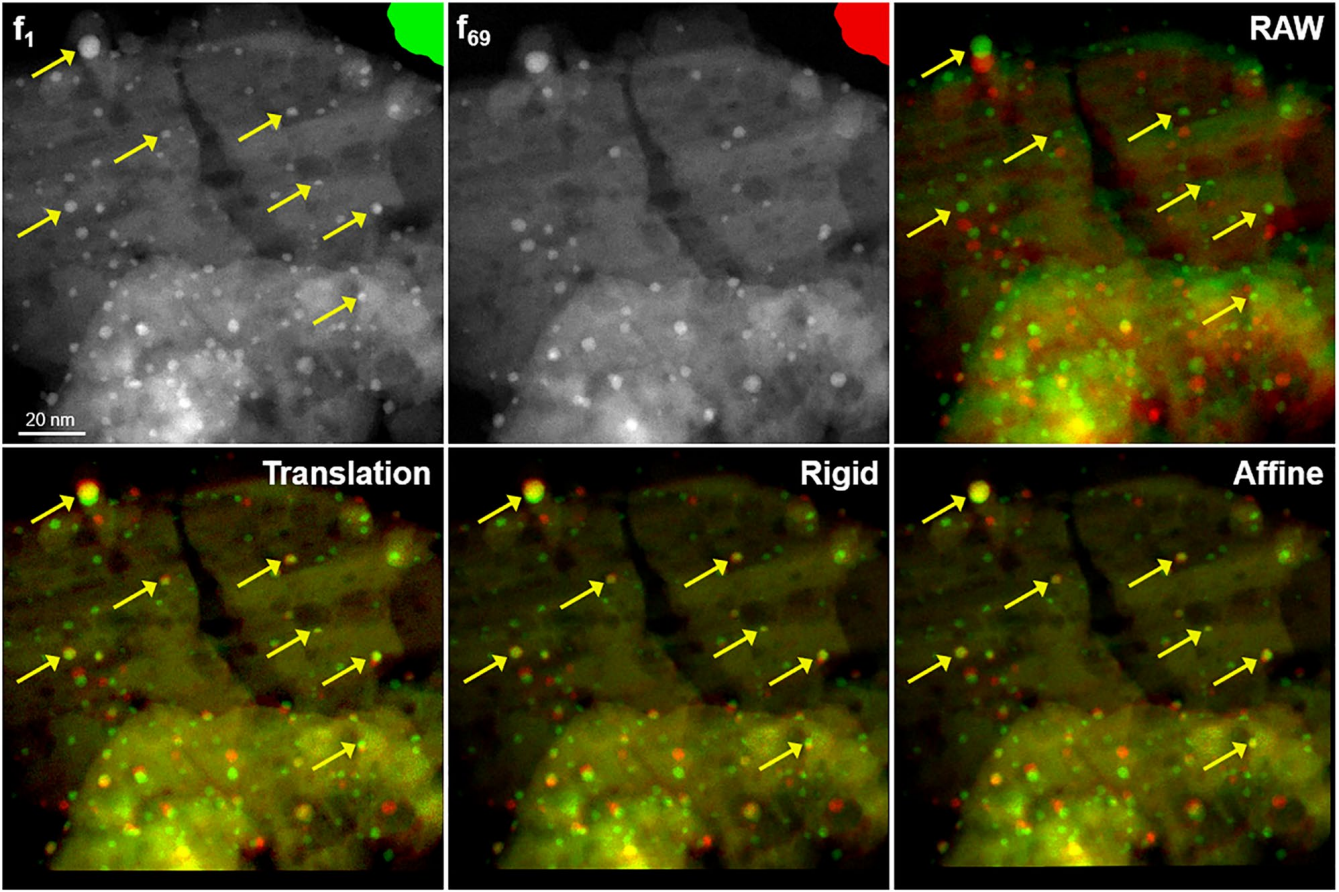
Registration results of 3 different transforms. Image $f_1$ is the reference image for all registrations. $f_{69}$ is the last image of the sequence. Colored images are merged channels of $f_1$ (green channel) and transformed $f_{69}$ (green channel) that allow a visual assessment of tested registration transforms: well-aligned areas are rendered in yellow. The alignment of the support on the whole image motivates our choice of the transformation type independently of the motion of particles from green to red dots. Yellow arrows point out some non-moving particles.

The specificity of in situ TEM imaging means that consecutively acquired images do not allow the exact same field of view to be observed. This scene is subject to different distortions (in practice, due to scan instabilities producing ‘shears’ in the successive lines of the image) and displacements (e.g. drift of the object) during acquisition. These perturbations directly influence the relative position of the particles observed in the image. Thus, to study precisely the path of the particles during the whole acquired sequence, it is necessary to remove these biases relative to the image shifts and not to the movements of the particles. Image registration is needed to reduce these shifts as much as possible using the first image of the sequence as a baseline. Many registration approaches exist^62–64^. Here we apply iterative registration methods well-used in a medical image context and which allow robust alignment of different kinds of image together with rigid constraints. The pipeline of the registration used is illustrated in the Supplementary Material (Supplementary Information - S.I. - file). The registration method consists in taking a reference image on which the other images, the moving images, of the sequence will be registered. Such image registration approaches consist in optimizing iteratively the parameters of a transformation *T* deforming an image on the reference image in order to maximize the similarity between the two images. Among the plethora of available similarity measures, we opted for maximizing the correlation coefficient between images^65^. The correlation coefficient is normalized by the auto-correlations of both reference and transformed images. From the present experiments, this normalized correlation proved to be perfectly robust to the different series processed contrary to the transformation used. We have studied 3 types of transformation likely to more or less address the deformations observed in the TEM image sequences: translation, rigid, and affine transformation. These alignments were first performed on a test sequence labeled ’Pd20’ recorded at room temperature (20$^\circ$C) and under high vacuum (9 10$^{-07}$ mbar) on the Pd(O)@$\delta$-Al$_2$O$_3$ system (see supplementary video Video02.avi; illustrations and a more complete analysis are also reported in the Supplementary Material, section SI-A). These experimental conditions were chosen to verify that in the absence of thermal stimuli and reactive atmosphere around the sample, the NPs stay immobile as expected if no motion is induced by some beam effect. It is shown that the affine method gives good results and, at the same time, confirms that the NPs are poorly mobile under the unique action of the electron beam at room temperature and under high vacuum. The same registrations routines were applied to the experimental series Pd250, see Fig. 3 and the supplementary video Video01.avi. In this case, the association of NPs at the beginning and the end of the sequence is rather tedious manually because the number of NPs evolves and several NPs move significantly. This will be shown later through the automatic tracking of their complete trajectories. Therefore, Fig. 3 simply illustrates visually the shifts obtained with the different transformations, considering a subset of manually selected non-moving particles. To make the registration step robust to our different data, a commonly used multiscale pyramidal approach with 5 resolutions was used^62^. Furthermore, we produce final registered images with a linear image intensity interpolation^62^. This interpolation method, which affects the intensities of the image, does not modify significantly neither the average values nor the integrated intensities of the particles.

From the top right image of Fig. 3, we observe that an image alignment is necessary. As for the Pd20 series described as Supplementary Material, the affine transformation (bottom right image) provides a good alignment and offers sufficient freedom to correct the intrinsic discrepancies in the acquisition. Note that the color mixing enhances the yellow intensity of rather immobile NPs, the initial and final positions of which are almost superimposed (yellow arrows).

#### Training U-Net with simulations

**Figure 4:**
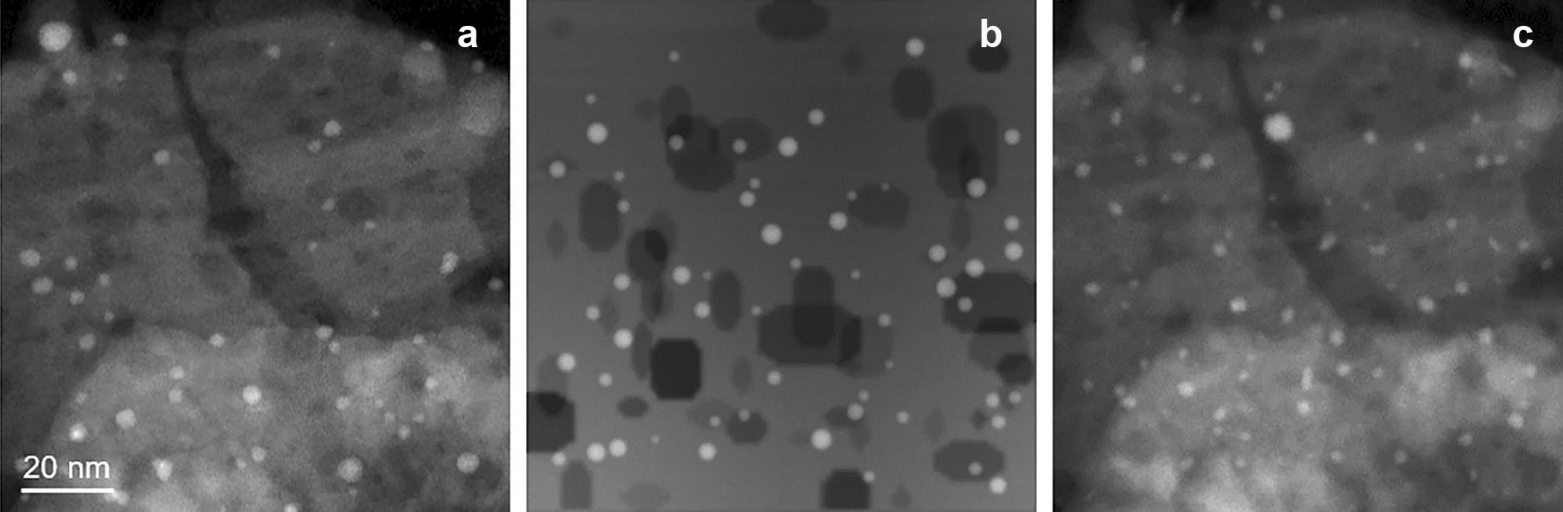
Experimental (**a**) and simulated images (**b**) and (**c**) related to the system Pd(O)@$\delta$-Al$_2$O$_3$. (**b**) is a simple simulation where the variations of the background intensity and dark areas respectively mimic thickness variations and the presence of pores in the support. (**c**) is deduced from the image (**a**) after erasing the NPs and generating new pseudo-circular ones.

STEM imaging relies on incoherent scattering, which can be reasonably modeled in a simple way to obtain realistic image simulations of microstructures consisting of a population of nearly spherical particles supported on a more or less porous substrate with a smoothly varying topography (see details in the Methods section). In a first step, U-Net was trained on numeric images generated by modeling both NPs and supports. For a fine-tuning procedure, simulated NPs were further generated on micrographs extracted from the experimental series over which the NP segmentation is to be performed, after having ‘Photoshop’-erased the real NPs to produce a clean support. Figure 4 illustrates both types of simulated images for the Pd(O)@$\delta$-Al$_2$O$_3$ catalytic system consisting of Pd oxide NPs on a delta-alumina support (see the Supplementary Information for more illustrations). Dynamic simulated sequences accounting for various interactions between NPs (see Methods and S.I. file) were further generated for testing the tracking procedures described in the next sub-sections. For generating an initial U-Net model, 2888 simulated images are used. 90 % of these serve as the training dataset, whereas the rest are reserved for the purpose of validating the performance of the model being trained. This U-Net model, consisting of 5 levels and having just over 30 million trainable parameters, was trained over images downsampled to a $256\times 256$ pixels resolution. This initial model was further refined through the fine-tuning procedure evoked above. According to various tests performed over 20-50 true support images, 20 images turned out to be sufficient for the optimal fine-tuning of the initial model.

#### Particle tracking

**Figure 5:**
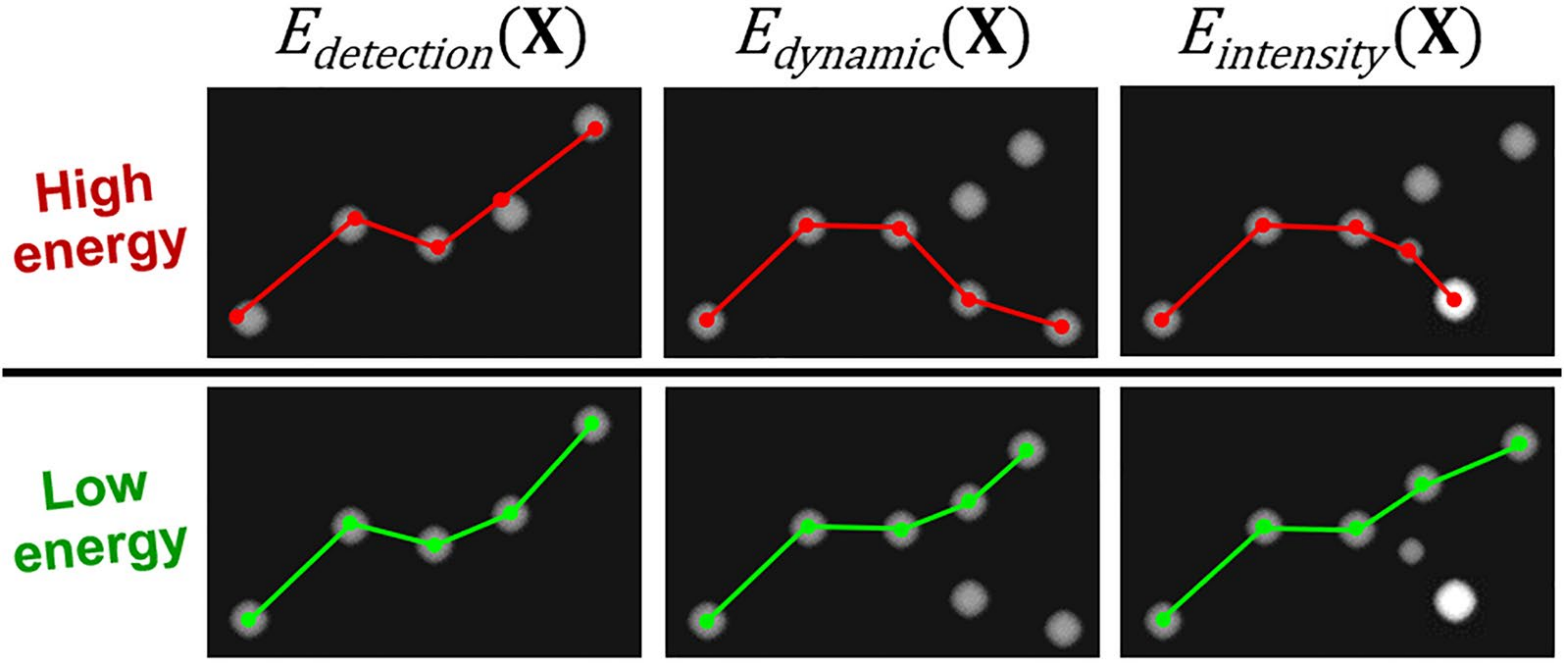
Illustration of the effect of the three main energy terms: the detection term keeps the trajectory close to the detected NP positions, the dynamic model term promotes short displacements and the intensity term enables mass conservation (figure inspired from^56^).

Depending on the application and the imaging system, the tracking of multiple objects can be more or less difficult due to the variable number of objects in the field, the possible overlapping of object images, possible occlusions or regularity of the trajectories. In our case, the behavior of nanoparticles is closely related to their physical properties. The typically expected behavior of a particle is to follow a Brownian motion and to grow due to Oswald ripening. The motion should decrease as the size of the particle increases and small mobile particles could be eaten by larger ones if they are close to each other. This later event can be denoted particle fusion. However, the particles being located on the surface of the substrate, if two particles appear close to each other, they can also be located on different sides of the substrate, typically above or below the support and thus their trajectory can cross without being able to fuse. Additionally, very small particles can be invisible at the beginning and become visible (birth) at a specific time due to growing. At last, a particle can be no more visible if it falls into a pore of the substrate. In summary, the total number of trajectories is not known and trajectories can start, stop or fuse each other at any time. Most existing tracking algorithms are using online greedy approaches. Starting from the detections in the first frame, they incrementally extend each trajectory by finding a set of associations between frame t and frame t+1 which minimizes a local criterion. However, this approach is unable to account for the complex behavior of the nanoparticles. Our approach is based on a global minimization algorithm denoted NP-Tracker and inspired from CEM-Tracker^56^, and specifically adapted to TEM images. This algorithm considers all the detections at all times and finds the best set of trajectories given the detections and their intensity. Formally, the set of trajectories is represented as a state matrix $\mathbf {X}$ giving the location $x_i^t$ of each target *i* at each time *t*. An energy function $E({\mathbf {X}})$ is defined to penalize states which are not coherent with the observed particles and the physical constraints. The minimization lies in finding the state matrix which minimizes $E({\mathbf {X}})$. The energy is composed of three main terms. A detection term to keep the solution close to the detections, a dynamic model term to favor weak displacements and an intensity term to provide intensity conservation. This latter term corresponds to the mass conservation of particles. It is particularly important to disambiguate situations with close trajectories where we can confuse two particles or where two particles are fusing. In this latter case, the resulting particle should have a final intensity obtained by summing the original particle intensities. The exact formulation of each energy term is given as supplementary information (section SI-C of the S.I. file) and an illustration of the effect of the three main terms is given in Fig. 5.

The resolution of the minimization is not trivial, first because the energy function is not convex and second because the dimension and the structure of the state matrix are not previously known. Typically, the number of columns of the matrix can vary according to the number of trajectories. Thus, according to the principle proposed in Milan et al.^56^, two alternate steps are applied to find the solution. First, a standard conjugate gradient descent is applied to locally converge to a minimum. Second, the dimension of the space is changed by applying structural transformations to the trajectories. Examples of structural transformations are adding, removing, splitting or merging trajectories. Another important part of our tracking algorithm is to provide both diameter and integrated intensity estimations for each detected NP enabling us to monitor their evolution along each trajectory. The diameter is classically estimated as the diameter of the disk having the same area as the segmented NP. The integrated intensity is estimated in two steps: (i) we compute the local background value $z_b$ as the mean value of the pixels surrounding the NP and (ii) we integrate the difference $z-z_b$ (*z* being the pixel value) over all the pixels inside the NP.

#### Validation of the approach on simulated sequences

**Figure 6:**
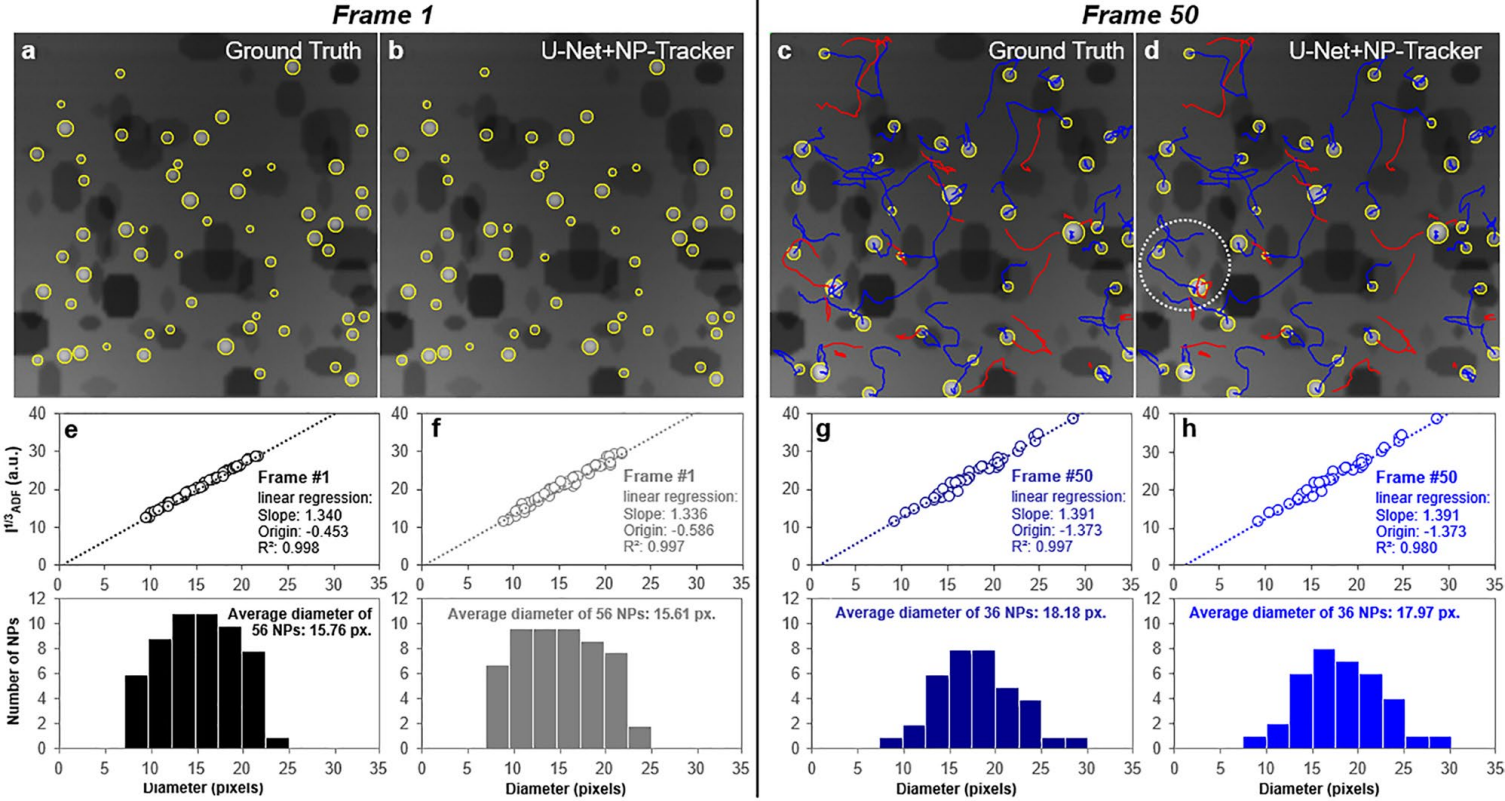
Results of the analysis of a simulated sequence (images 256x256 pixels representing a field of view of 100x100 $nm^{2}$). (**a**, **b**) Comparison of the Ground Truth (**a**) and automatic (**b**) analyses for the U-Net detection of NPs (yellow circles) at the beginning of the sequence (first frame). (**c**, **d**) same as (**a**, **b**) at the end of the sequence after 50 frames during which NPs have experienced a random walk with an average targeted displacement of about 1-2 pixels between successive frames. Ongoing trajectories are shown in blue, whereas ended ones are displayed in red. (**e**–**h**) Similar presentation for quantitative NP diameter results (in pixels) respectively at the beginning (**e**, **f**) and the end (**g**–**h**) of the sequence: Treacy-Rice analysis (see text and Methods section for details) on the top and size histogram below.

Before being used to analyze a real sequence, NP-Tracker has to be thoroughly evaluated. For that purpose, we can rely on the simulations’ algorithm previously used to generate the U-Net training images. A specific module has thus been added to simulate sequences of moving particles where their dynamics are more or less compatible with a Brownian motion. Various simulated sequences of moving NPs have been analyzed by the previously described two-steps procedure: U-Net-based segmentation of NP positions and NP-Tracker reconstruction of their trajectories. In each case, the exact position, size and intensity of each NP across time is perfectly known and this Ground Truth information can be compared to the estimations given by NP-Tracker. Figure 6 illustrates the analysis of a short sequence of 50 images containing a moderate number of moving NPs, all on the same surface of the support. (this example is further documented by the supplementary videos Video03.avi and Video04.avi and analyzed in sections SI-D and E of the S.I. file). Several metrics can be used to evaluate the U-Net + NP-Tracker performances. Apart from the basic numbers of false positives (FP) and false negatives (FN), other values classically used in computer vision to evaluate tracking algorithms^66^ are also calculated: the number of identity switches (IdSwitches) and the Multi-Object Tracking Accuracy and Precision (MOTA and MOTP; see section SI-E in S.I. file for additional information on these parameters). Numerical values for the simulated series presented in Fig. 6 are reported in Table 1 which highlights the quality of the training.

**Table 1:** Values of evaluation parameters for the simulated series containing 56 trajectories over 50 frames with a total of 2213 NP positions to detect.

**Measures**	**Values**
**FP**	0
**FN**	9
**IdSw**	2
**MOTA**	99.5
**MOTP**	94.9

More detailed information about this simulated dynamic sequence is given in the S.I. file, which also reports a table of main input parameters. In particular, the occurrence of coalescence/fusion events has been made possible according to the following conditions: NP candidates must be closer than a critical distance and the fusion occurs randomly with a low and controlled probability. A close inspection of Fig. 6 reveals an excellent agreement between the results of the present analysis with ground truth data straightforwardly deduced from the simulations. At first, the trajectory analysis in Fig. 6b shows an excellent identification of the NP-Tracker approach (right-hand side in b) except in the encircled region where two trajectories being very close during a few successive frames have been interchanged. In addition, Fig. 6 illustrates that the routine measuring the integrated intensity of NPs $I_{STEM}$ after a local background subtraction works reasonably well since the expected linear variation of the Treacy-Rice relation $I_{STEM}^{1/3}$ = f(D) (where D is the projected diameter of any NP belonging to a chemically homogeneous population^67, 68^) is properly verified at each frame (only the first and last frames of the sequence are reported here). It is seen that NP intensities retrieved during the tracking step f) and h) are almost perfectly identical to the ones input in the simulation e) and g).

**Figure 7:**
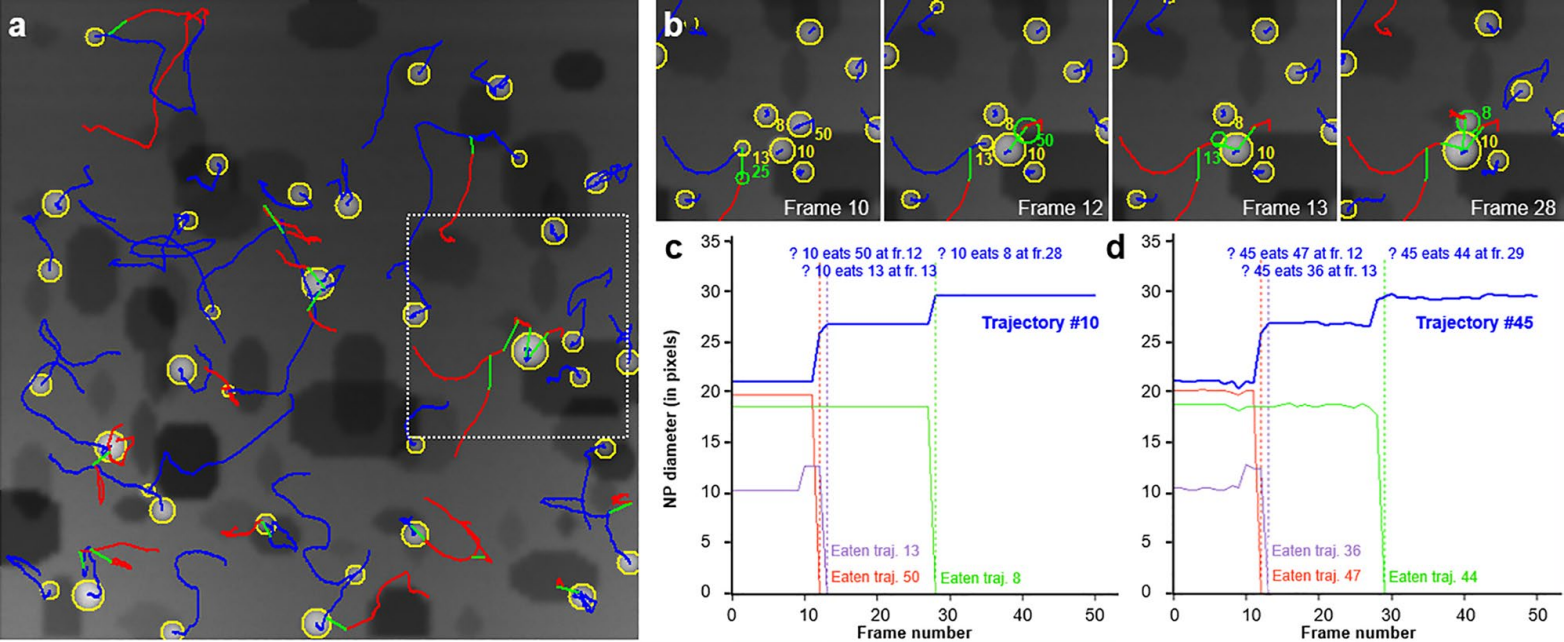
Description of the analysis of fusion events. (**a**) Last frame of the simulated sequence introduced in Fig. 6 and showing the last move of all ‘eaten’ NPs over all frames (green segments). (**b**) Enlarged detail of the area marked by the dotted rectangle in (**a**). NP are labelled as they were generated by the simulation program. (**c**, **d**) diagrams showing the evolution of the size of NPs concerned by the sequence involving the ‘eating’ NP # 10 as a function of frame number: ground truth (**c**) corresponding to (**a**, **b**) and NP-Tracker analysis in (**d**). Note that the NP-Tracker routine has generated different labels for the same NPs in (**d**) in comparison with the labelling of the simulation code (**a**–**c**).

An additional feature of importance in the interpretation of the trajectories is the identification of fusion events, as they have been produced several times in the simulated sequence. We have developed a routine to identify such events on the basis of identified trajectories after the NP-Tracker analysis. Three criteria more developed in the S.I. file have to be fulfilled to confirm a fusion: (i) one NP must disappear, (ii) another NP in its close vicinity must have grown in size and intensity, (iii) these changes must respect some mass conservation. Figure 7 reports a typical example of such a fusion analysis, demonstrating that it works nicely in the case of simulated data.

### Application to a real case

#### Real case tracking during in situ calcination of the Pd(O)@$\delta$-Al$_2$O$_3$ system

**Figure 8:**
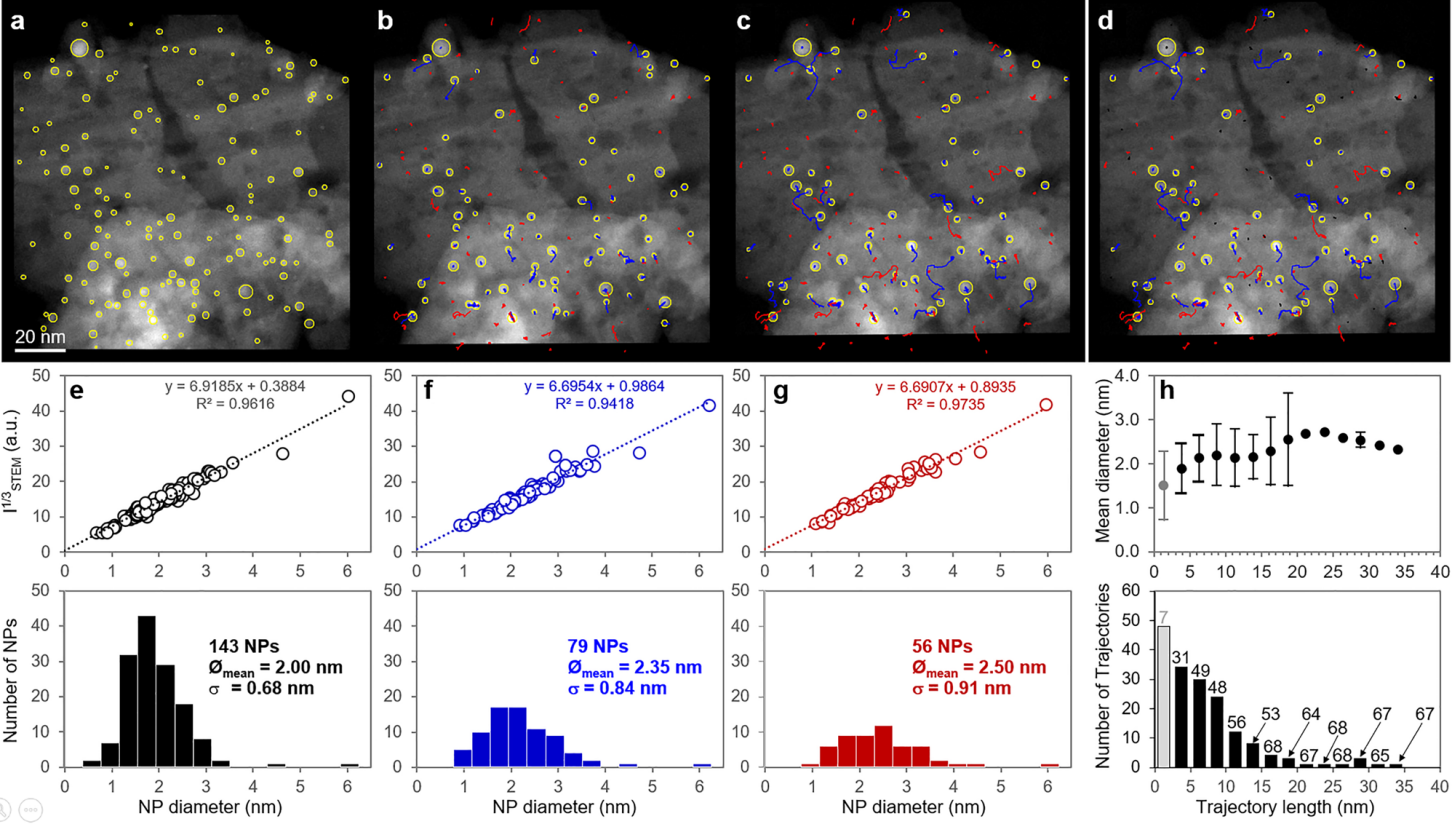
Results of the analysis of the Pd250 series. (**a**–**c**) illustrations of the automatic U-Net detection of NPs (yellow circles) and the NP-Tracker trajectory analysis respectively at the beginning, middle and end of the sequence after about 3 hours and 40 minutes. Ongoing trajectories are shown in blue, whereas ended ones are displayed in red. (**d**) Same as (**c**) but with the shortest trajectories (less than 2.5 nm, the mean NP diameter at the end of the sequence, see in f) shown in dark. (**e**–**g**): Quantitative NP size results corresponding respectively to frames (**a**–**c**) Treacy-Rice analysis on the top and size histogram below. (**h**) mean diameter of NPs as a function of the length of their trajectories (top) and histogram of trajectories lengths (bottom; numbers indicate the numbers of frames during which trajectories of the corresponding class are running). Both plots in (**h**) correspond to the end of the sequence.

In complement to the validation of our approach on simulated images, we have treated the experimental sequence Pd250 introduced in Fig. 3. Figure 8 summarizes its analysis.

Results look very consistent from both visual and numeric points of view. Treacy-Rice plots in Fig. 8d–f exhibit a reasonable linear behavior with little scatter, a very comparable slope at all times (indicating a chemical homogeneity) and a y-intercept close to zero as expected. Size histograms below illustrate the gradual decrease of the number of NPs and the slight size increase as indicated. Furthermore, additional plots in Fig. 8g refer to the analysis of trajectory lengths which will be commented on in the next subsection.

#### Application of the analysis for a better understanding of the evolution of an NP population

**Figure 9:**
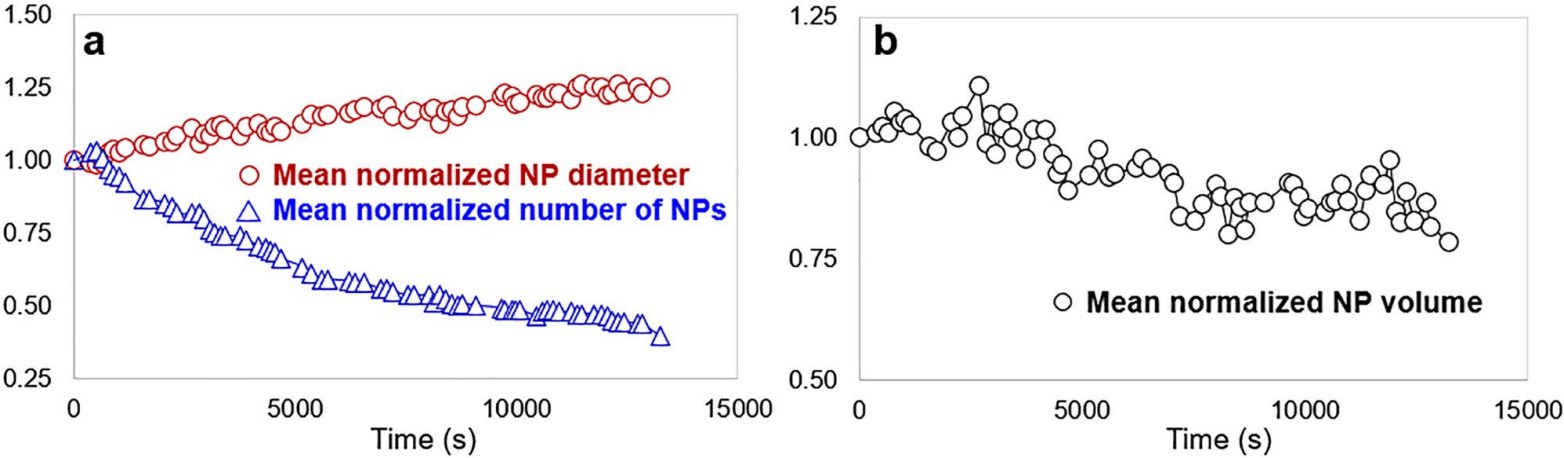
Quantitative evolution of the PdO NP population deduced from the U-Net / NP-Tracker analysis of the Pd250 series. (**a**) mean normalized diameter and number of NPs as a function of time. (**b**) Similar evolution of the normalized volume of all NPs.

Numeric data deduced from the previous analysis allow to quantify the dynamics of the Pd NPs during their evolution during the calcination process, as illustrated in Fig. 9. As can be seen from Fig. 9a, the NP size increases monotonically all along the sequence: this indicates that growth occurs without reaching a stationary state as ideally expected for avoiding any loss of potential catalytic activity. The final size after this sequence remains however reasonably small (2.5 nm starting from a mean value of 2 nm). The complete calcination sequence performed up to 450$^\circ$C leads to a PdO NP size of about 3.4 ± 0.5 nm: this treatment is moreover followed by a lower temperature treatment under hydrogen to reduce the PdO phase into metallic Pd, which reduces slightly the final Pd size^38^. Accordingly to the size increase, it is seen that the number of NPs decreases gradually. Another important feature (Fig. 9b) is that the total volume of particles decreases, meaning that there is a loss of matter. It is strongly believed that this diminution tendency is too large to be due to possible measurements errors, which nevertheless may explain the local departures from the mean curve. Although all curves exhibit a tendency to stabilize at the end of the sequence, it may be concluded that the loss of matter evidences a probable beam effect. All these features will be consistently reconsidered in the next section.

#### Interpretation of the NP detection and trajectory analysis

**Figure 10:**
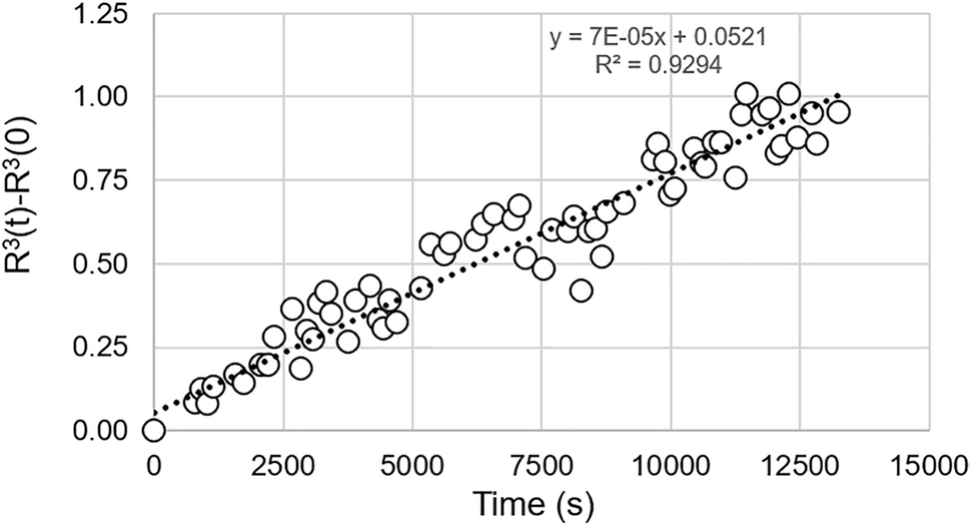
Plot of $R^{3}(t)-R^{3}(0)$ as a function of time for PdO NPs during the in situ ETEM calcination at 250$^\circ$C.

Previous features deduced from the combined and automatic U-Net/NP-Tracker analysis allow to quantify the behavior of PdO NPs during the ETEM in situ calcination. NP growth is definitely confirmed through the evidence of both the increase in size and the decrease of the number of NPs (Figs. 8 and 9a); see also the supplementary video Video05.avi). However, The decrease in the total volume of NPs as shown in Fig. 9b indicates that there is a noticeable loss of matter during the heating experiment. The estimation of the volume might be questionable since it relies on the assumption that NPs are spherical, but this assumption seems reasonable according to the consistency of the Treacy-Rice analysis as plotted in Fig. 8d–f). This behavior is certainly due to some sublimation induced by the electron beam, which leads to a matter loss. At the same time, this continuous shrinkage of smaller NPs may also contribute to a continuous atom flux towards larger NPs, which explains the monotonic size increase depicted in Fig. 9a. Irradiation effects were already observed during in situ ETEM experiments on the Pt/alumina system under oxygen where the acceleration of shrinkage of NPs while increasing the electron dose during ripening was demonstrated^35^. The evolution of the mean NP is plotted in Fig. 10 under the form where volume diffusion is the rate-controlling factor of the NP growth^69^:

$$\begin{aligned} R^{3}(t)-R^{3}(0) = Kt \end{aligned}$$

where *R*(*t*) represents the mean NP radius at time *t*, and *K* the rate constant encompassing physical parameters, among which the diffusion coefficient at the temperature of interest. In this relationship, the value of 3 for the exponent is the signature of a volume diffusion regime which may gradually change to an interface-driven regime as the temperature is increased as discussed by^37^).

The fact that the evolution of the Pd250 series with images recorded every 3 minutes follows properly the previous relation confirms the previous analysis: while inducing some loss of matter, the electron beam also favors the diffusion of atoms from smaller and vanishing NPs onto larger ones. Despite the undesirable beam effect, it can be stated that the NP growth proceeds by a classical thermodynamical mechanism.

## Discussion

**Figure 11:**
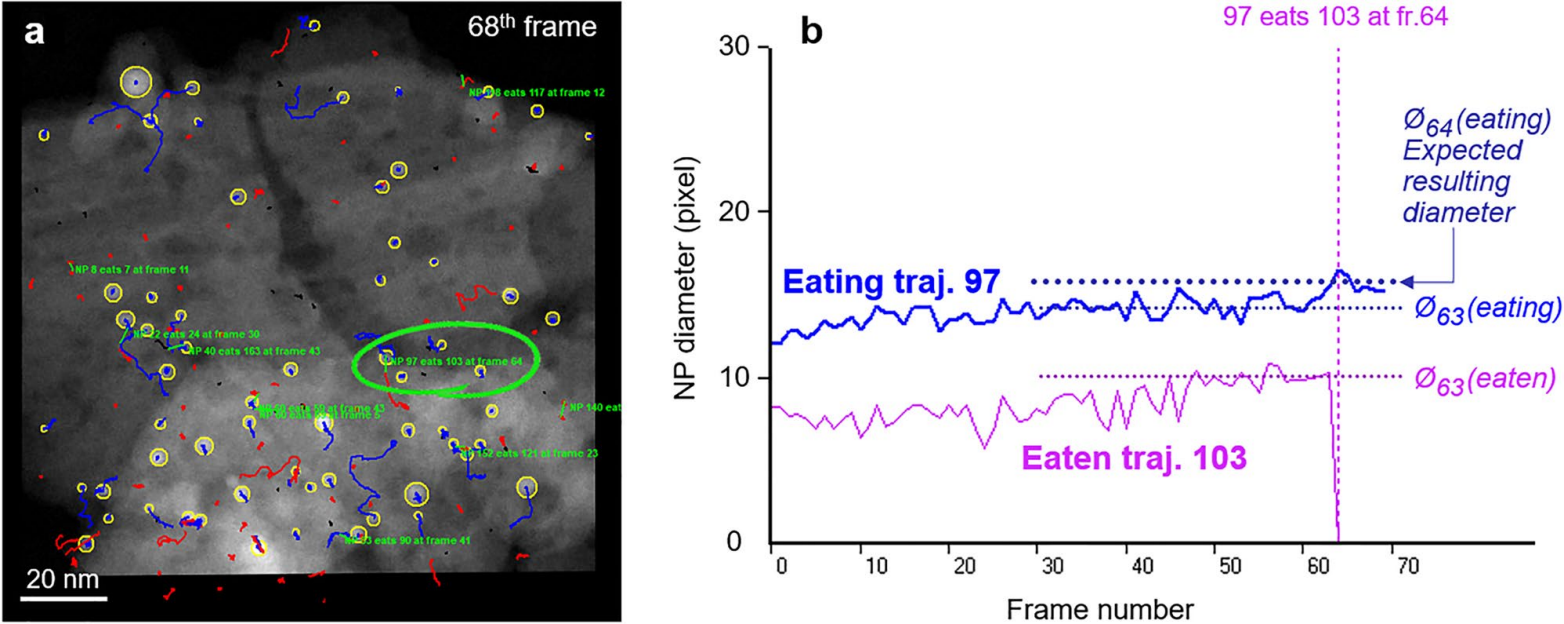
automatic analysis of fusion events. (**a**) Last frame of the Pd250 series with the indication of identified fusion events (green text). (**b**) plot of the evolution of NP diameters Ø(97,f) and Ø(103,f) as a function of time (frame number f) for the event encircled in (**a**) where NP (trajectory) 97 absorbs NP 103 at frame 64. Horizontal dotted lines visualize the value of the ‘eaten’ and ‘eating’ NP diameters at frames 63 and 64 (respectively just before and after the fusion); the upper line marks the expected value of the diameter of the resulting NP which corresponds to the automatically measured increased diameter.

The reliability of the NP detection results reported for the experimental data shown in Fig. 8 appears to be very satisfactory and it has been confirmed that U-Net leads to significantly a better detection than what can be obtained through a classical image processing based on a local threshold or correlation-based approach as demonstrated by the analysis reported in the S.I. file, section F. In addition, the detection appears to be relatively efficient regarding the noise since NPs exhibiting a low signal-to-noise ratio are detected with a much better efficiency than what can be expected from a local threshold-based image processing (see a more detailed analysis and discussion in the S.I. file, section G). Although Fig. SI-F3 and Table SI-T2 show that lost smallest NPs, due to their low SNR, could be retrieved by a correlation-based local approach, a more drastic increase of flawed FP detections is unfortunately induced. Improving properly such detections would indeed require to increase the acquisition time, but at the expense of significant irradiation damages and loss of temporal resolution, with a great impact on the accuracy of the trajectories reconstruction. Furthermore, results presented in the previous section allow discussing the mechanisms of NP growth under the present experimental conditions. The analysis above can be achieved by considering numeric data deduced from the analysis of trajectories, as reported in Fig. 8g. The histogram at the bottom shows that a large number of NPs remain almost immobile (first class near zero). The graph at the top further indicates that this class of NPs has the smallest average size, around 1.5 nm. Although it may appear counterintuitive that the smallest NPs are the immobile ones, it can easily be explained by the fact that these NPs vanish very rapidly and do not survive a long time. Numbers reported on top of each trajectory class in the histogram of Fig. 8g indicate the average number of frames during which the trajectories of the corresponding class exist: the first class corresponds to 7 frames, meaning a very fast disappearance of the associated NPs. These trajectories are thus not representative and are displayed in gray. Interestingly, the size of moving NPs increases when their mobility increases (thus experiencing longer trajectories) but decreases for the longest trajectories, consistently with the expected behavior of higher mobility for small NPs. Note that these latter objects are moving during almost the whole sequence (numbers of frames close to the maximum of 69). Although these features can reasonably be explained, they must be considered with care because the statistics for each class, especially for those corresponding to the longer trajectories, remain very poor with one object per class in several cases. As previously mentioned, the tracking of trajectories allows identifying fusion events, as illustrated in Fig. 7. Figure 11 summarizes this analysis: only 10 fusion events have been detected over a total number of 143 NPs initially detected and 171 trajectories, among which 123 significant ones with a total length greater than 2.5 nm (the upper limit of the first class in the histogram of Fig. 8g. This means that aggregative growth of NPs takes place along only $8 \%$ of the trajectories, confirming that Ostwald Ripening is the main operating process leading to the size increase during the calcination treatment. Despite the consistency of this analysis, it must again be considered with care, since any possible events occurring during the ’beam off’ period in between successive images are obviously not recorded.

## Conclusions

This work has reported a deep learning and computed vision combined approach to analyze quantitatively the evolution of a population of supported NPs during in situ ETEM experiments under reactive conditions in the context of heterogeneous catalysis. The calcination of the palladium (oxide) / delta-alumina system was used to illustrate the methods that have been developed and the type of results that can be deduced. The main conclusions of this study are as follows:

- Recorded video-type sequences of STEM micrographs in ETEM allow to track the nanoparticle trajectories during their dynamic evolution on a heterogeneous support with contrast variations, subject to a proper registration of the image series as it was performed with the affine alignment procedure.
- The U-Net neural network is well adapted to the detection of supported nanoparticles exhibiting an extra intensity with respect to the background in STEM images. It can beneficially be trained on simulations generated according to the ’Z-contrast’ power law in STEM.
- Both trajectories and fusion events have been correctly retrieved by dedicated routines in the NP-Tracker algorithm, as attested by the excellent values obtained when applying multiple objects tracking evaluation metrics.
- When applied to the experimental data, our automatic analysis provides statistical results regarding the size and the trajectories of PdO nanoparticles, together with a quantitative evaluation of fusion events which allows to evaluate the balance between Ostwald Ripening and direct aggregative coalescence to explain the measured size increase. For the system studied here, coalescence remains a minor growth mechanism.

Further dynamic sequences recorded on other systems in different ETEM conditions have already been treated or are still under investigation; they will serve to improve the quantification of specific events such as NP crossings and to work on a better machine learning integration on both steps, i.e. nanoparticle detection and trajectories analysis.

## Methods

### Environmental TEM (ETEM)

Experiments were conducted in situ in a dedicated FEI Titan ETEM operated at 300 kV. The catalytic system Pd(O)@$\delta$-Al$_2$O$_3$ chosen for illustrating the method consists of Pd oxide NPs supported on porous delta-alumina grains. Crushed powders were deposited on heating nanochips mounted on a Wildfire specimen holder from DENSsolutions. The in situ calcination was performed under an oxygen partial pressure typically of a few mbar up to 450$^\circ$C. During these sequences, the dynamic evolution of the NP under gas and in temperature was followed by recording periodic Annular Dark Field STEM images (ADF-STEM, $2048\times 2048$ pixels, dwell time 10 $\mu$s - full image scan about 42 s) with different constant time intervals of a few minutes in order to evaluate the effect of prolonged exposure to the electron beam. It is important to notice that we choose to acquire STEM images instead of conventional bright-field TEM images: although the latter should provide a better temporal resolution, (i) they provide the worst conditions for the detection of NPs (due to diffraction contrast) and (ii) they are more likely to induce irradiation damages^38^. Furthermore, our STEM imaging conditions should allow to image very similarly NPs lying on the top or bottom surfaces of the supporting alumina platelets, or at any height considering their topography. These platelets are indeed quite thin: typically 10 nm as demonstrated by tomography experiments^38^. This thickness is typically less than the depth-of-focus *T* of STEM images when recording with a conventional, not probe corrected, illumination - e.g. *T* equal to 20 nm in a Titan TEM at 300 kV using a probe convergence half angle of about 10 mrad^70^ -. We report here results obtained on a ’Pd250’ sequence of images recorded every 3 minutes with a ’Beam off’ time interval of less than 1 minute during about 3 and a half hours under 2.2 mbar of oxygen at 250$^\circ$C (see the supplementary video Video01.avi). Other ETEM sequences recorded on different areas under the same temperature and gas conditions^38^ but with increased ’Beam off’ times show a significantly lower NP mobility as compared to the present case where a shorter time interval was used (this earlier work further documents the sample synthesis, TEM preparation and experimental details).

### Simulated images for U-Net training

In a reasonable approach, the ADF-STEM scattered intensity by a thin sample can easily be described by the following power law:

$$\begin{aligned} I_{STEM} = C \sum _{k} n_k Z_k^{\alpha } \end{aligned}$$

where:

- *C* is a constant describing related to experimental conditions such as ADF detector efficiency, beam current, acquisition time (omitted in the following),
- $I_{STEM}$ is the intensity collected on an annular dark field detector positioned under the sample scanned by a focused electron probe,
- $n_k$ is the number of atoms of the specie k present in the probed volume at each position of the probe, with $Z_k$ their atomic number,
- $\alpha$ is an exponent slightly less than 2, the exact value of which ranges between 1.3-1.8 according to the geometry of the ADF detector (i.e. its range of collection angles).

Neglecting beam broadening effects and more complex interactions in sufficiently thin samples with an ideally low mass-thickness allows writing directly:

$$\begin{aligned} I_{STEM} = V \sum _{i} \rho _i Z_i^{\alpha } \end{aligned}$$

where:

- *V* is the probed volume proportional to the local sample thickness assuming the probe size remains constant,
- $\rho _i$ is the atomic density in number of atoms per unit volume for each chemical species *i*.

Beyond the generation of static images, dynamic sequences were also generated according to a homemade routine written in Visual Basic (Microsoft VB6) with controlled features mimicking the experimental behavior. On one hand, these features include amplitude and direction of NP displacements (parameterized to account for possible variations of their speed of motion as a function of their sizes or the local topography of the support). On the other hand, they also incorporate events such as fusion (coalescence) of NPs with a variable probability of occurrence as illustrated in the S.I. file, crossing of superimposed NPs possibly deposited on the upper and lower surfaces of the support and disappearance or appearance of (small) NPs. Other VB6 routines were also generated for an adequate display of the trajectories and a post-mortem analysis of fusion events (see S.I. file). Typical relevant parameters for a simulated sequence of numeric images resembling an experimental series for the Pd(O)@$\delta$-Al$_2$O$_3$ system are reported in the S.I. file (sections SI-D and E). Interestingly, the consistency of both simulated and experimental images is well demonstrated using the method developed by Treacy and Rice^67,68^, see Fig. 6c,d.

## Supplementary Information

Supplementary Information 1.

Supplementary Information 2.

Supplementary Information 3.

Supplementary Information 4.

Supplementary Information 5.

Supplementary Information 6.

## Data Availability

Raw TEM data from which supplementary videos have been created are available from the corresponding author upon reasonable request. Computer codes are part of an ongoing research project still in progress and will not be available before the end of 2022.
