# Supplementary material for: Deep learning detection of nanoparticles and multiple object tracking of their dynamic evolution during in situ ETEM studies
Source: Sci Rep. 2022 Feb 15;12:2484. doi: 10.1038/s41598-022-06308-2 (PMC8847623; doi:10.1038/s41598-022-06308-2)
Supplement: Supplementary file 1 — Supplementary Information 1. [file 41598_2022_6308_MOESM1_ESM.pdf]

Supplementary Information

Deep Learning detection of nanoparticles and multiple object tracking of their dynamic evolution during in situ ETEM studies

Khuram Faraz[1,2], Thomas Grenier[3], Christophe Ducottet[1] and Thierry Epicier[2,4*]

[1]Université Lyon, UJM-Saint-Etienne, CNRS, Institut Optique Graduate School, Laboratoire Hubert Curien, UMR5516, 42023 Saint-Etienne, France
[2]Université Lyon, INSA-Lyon, Université Claude Bernard Lyon 1, CNRS, MATEIS, UMR 5510, 69621 Villeurbanne Cedex, France
[3]Univ Lyon, INSA-Lyon, Université Claude Bernard Lyon 1, CNRS, CREATIS, UMR 5220, INSERM U1206, 69621Villeurbanne Cedex, France
[4]Univ Lyon, Université Claude Bernard Lyon 1, CNRS, IRCELYON, UMR 5526, 69626 Villeurbanne, France

[*]corresponding author, thierry.epicier@ircelyon.univ-lyon1.fr

In addition to the supplementary information provided in the following sections, several videos have been added:

- **Video01.avi**: series of 69 STEM images recorded every 3 minutes during an in-situ calcination experiment of the Pd(O)@δ-Al_2O_3 system at 250°C under 2.2 mbar of oxygen; both raw pre-alignment (left) and optimized affine alignment (right) are shown.
- **Video02.avi**: series of 64 STEM images recorded every 3 minutes at room temperature (20°C) and under high vacuum (9 10^{-07} mbar) on Pd(O)@δ-Al_2O_3 and commented in the SI-B section below.
- **Video03.avi**: simulation of a dynamic sequence commented in the SI-D and E sections. The green annotations indicate fusion events respectively identified manually and automatically in the ground truth and U-Net + NP-Tracker cases respectively.
- **Video04.avi**: Quantitative measurements performed on all successive frames of the simulated sequence: Treacy-Rice plot of integrated NP STEM intensity $I_{STEM}^{1/3}$ vs. NP diameter (in pixels) and NP size histograms.
- **Video05.avi**: U-Net and NP-Tracker analysis of the Pd250 series; the raw pre-aligned series is recalled (left).

SI-A. Image registration pipeline

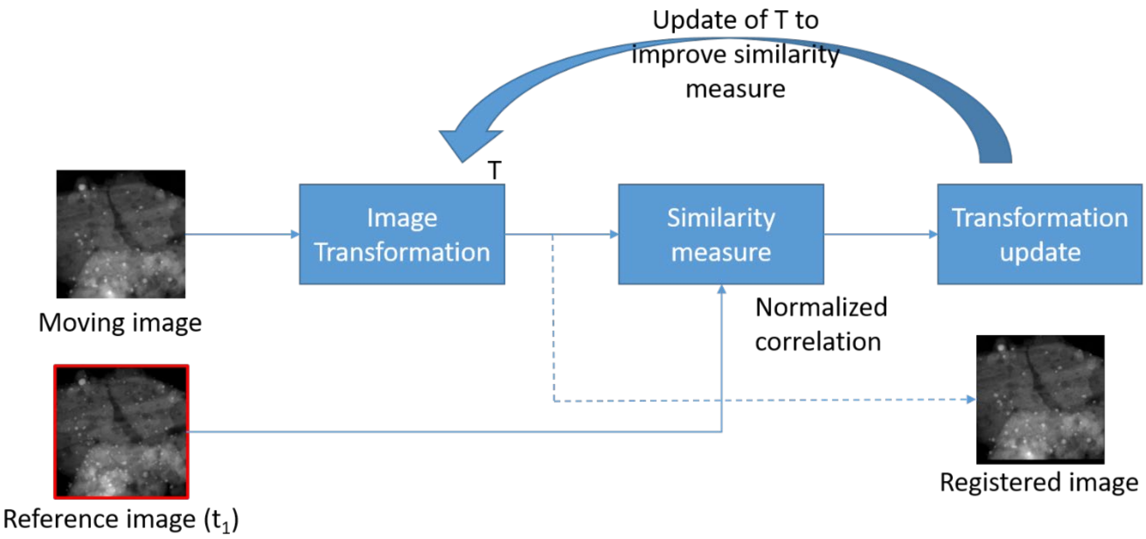

Figure SI-A1: Registration pipeline. The input image to be registered is the moving image. The transformation parameters are updated iteratively to improve similarity between the reference image and the transformed moving image. When we observe no more improvement of the similarity measure, the transformation T is said to be optimal and allows the transformation of the moving image to produce the registered image. Interpolations steps are not represented in this figure.

SI-B. Experimental STEM sequence Pd20 (20°C, 9 10^{-7} mbar)

The STEM series (see supplementary video Video02.avi) consists in 64 micrographs acquired every 3 minutes during about one hour and prealigned classically by cross-correlation leading to the starting 'raw' state. 3 selected registration methods have then been applied translation, rigid, affine. Finally, the positions of all NPs in all frames were determined by application of the U-Net detection procedure, and their trajectories were identified with NP-Tracker as described in the main text. From this analysis, the displacements [dx,dy} of all NPs in the last frame with respect to their initial positions in the first frame were plotted as shown in Figure SI-B1. For each diagram plotted in this figure, the barycentre of all NPs in the last frame is also plotted in the (x,y) space where the corresponding barycentre in the first frame is set at the origin.

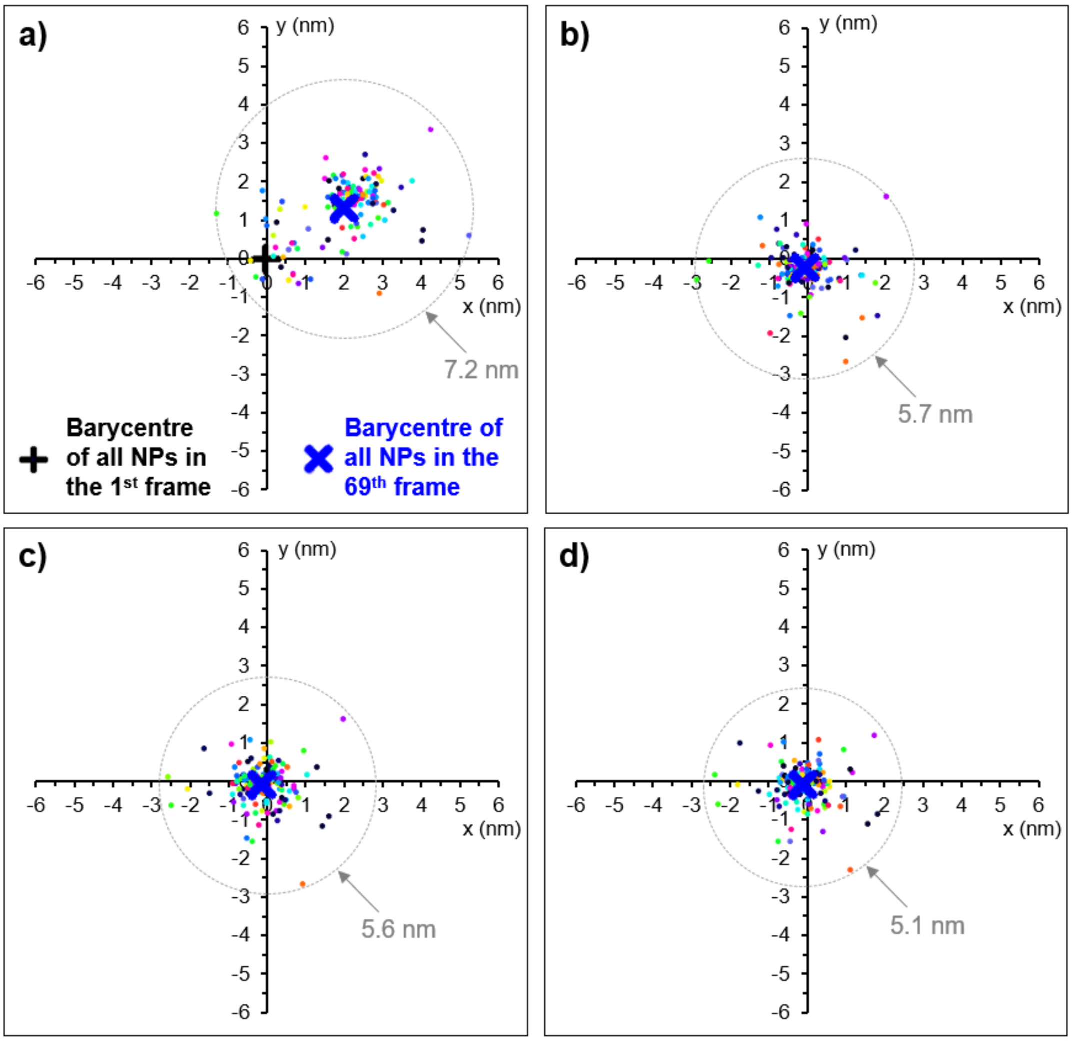

Figure SI-B1: Analysis of NPs positions with various registration methods for the 'Pd20' STEM series. a): 'Raw' pre-alignment by cross-correlation of the whole images. Color dots show the displacements {dx,dy} of all NPs in the last frame with respect to their initial position in the first frame. Note that their barycentre at the end of the sequence has drifted with respect to the origin; the grey circle includes all NP displacements. b-d): Similar plots for the translation, rigid and affine alignments respectively. In each of the 2 latter cases, the final NP positions are found very close to their initial ones with a barycentre very close to the origin.

The improvement of the affine alignment after a rough cross-correlation is demonstrated by figure SI-B2. IT is clearly seen that in the absence of temperature and/or non-inert atmosphere, Pd NPs remain essentially immobile.

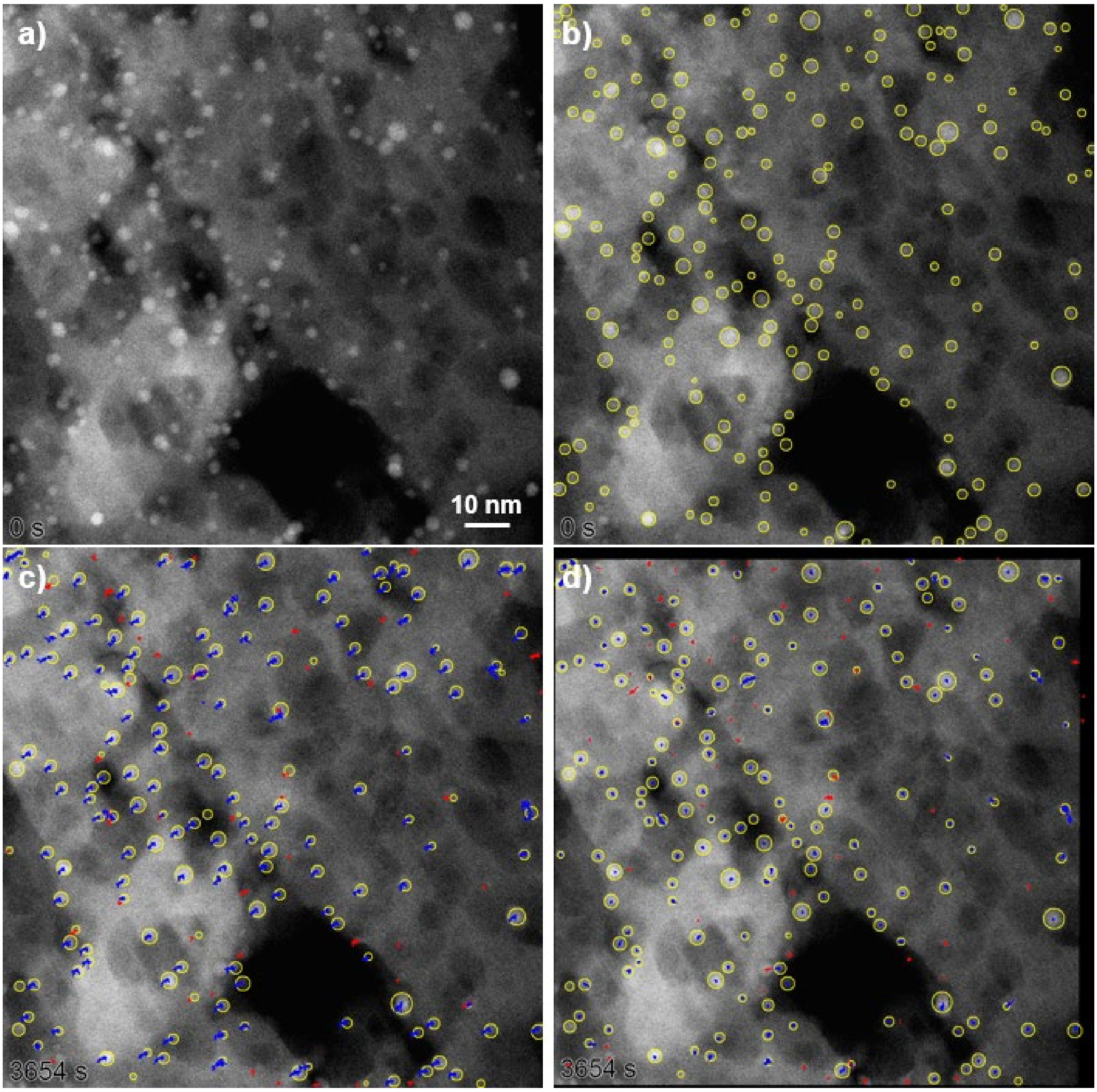

Figure SI-B2: Display of NP positions and trajectories for the 'Pd20' STEM series. a-b): First ‘Raw’ image of the series with markers identifying the NPs in b). c-d): Display of trajectories (blue segments, red markers refer to trajectories that have stopped in preceding frames) in the last frame for the 'Raw' and ‘affine-aligned’ series respectively. Note that trajectory markers in d) are almost punctual.

Finally, the statistical analysis of the NP population (i.e.: Treacy-Rice analysis – see Methods section for more comments – and size histograms) as reported in figure SI-B3 confirm this finding.

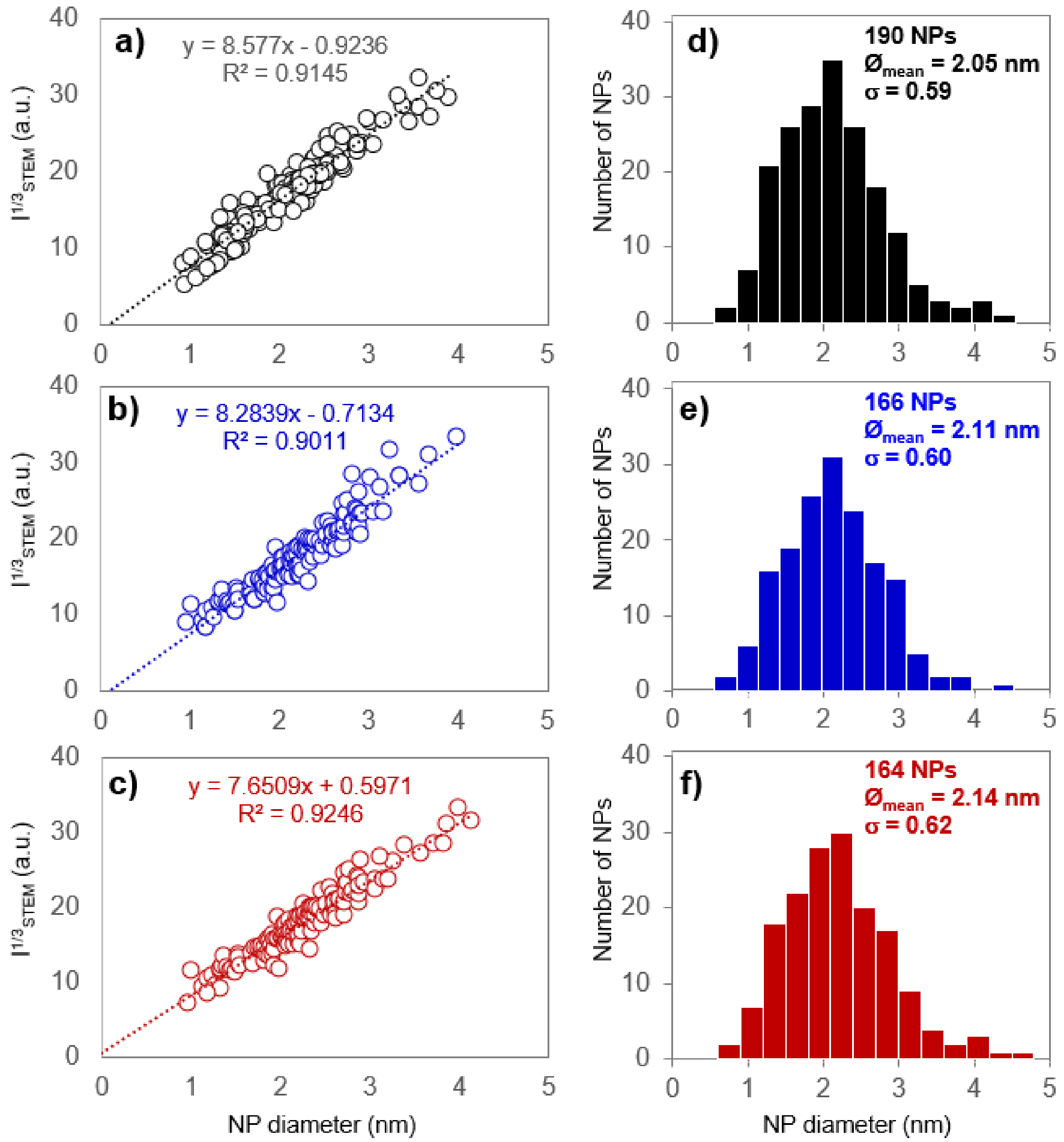

Figure SI-B3: a-c): Treacy-Rice analysis respectively of the start state, final state after a 'Raw' alignment and final state after an affine alignment. d-f): Similar to a-c) for the size histograms. These data show very little changes from the start to the end of the sequence due in particular to the quasi-immobility of NPs.

SI-C. Energy model used in NP-Tracker

Principle and notations

At the end of the detection step, for each frame $t \in \{1,...,F\}$ (F is the number of frames), we have detected a set of candidate NPs. We denote $D(t)$ the number of detected candidates in frame t and we denote g the index of each detection. The (X,Y) location of detection g in frame t is then denoted $\mathbf{D}_g^t$.
The goal of the tracking is to determine the best set of trajectories according to the detections. Formally, if i denotes the index of a solution trajectory, we have to determine the state vector $\mathbf{X}$ which consists of the (X,Y) coordinates of each NP along their solution trajectories. We suppose that a given trajectory i exists only over the set of frames $t = s_i,...,e_i$, s_i being the first frame where the corresponding NP exists and e_i the last one. The general term of the state vector to determine is thus $\mathbf{X}_i^t$ with $i = 1,...,N$ and $t = s_i,...,e_i$.

According to the method proposed by Milan et al. [1], the principle is (i) to define an energy function $E(\mathbf{X})$ giving how well the state vector fits the detections and fulfill the physical constraints and (ii) formulate the problem as the minimization of this energy function i.e. find the state $\mathbf{X}^*$ which globally minimize the energy function:

$$\mathbf{X}^* = \arg\min_{\mathbf{X}\in\mathbb{R}^d} E(\mathbf{X})$$

Energy terms

The energy function is a linear combination of five individual terms:

$$E = E_{\text{det}} + \alpha E_{\text{int}} + \beta E_{\text{dyn}} + \gamma E_{\text{exe}} + \delta E_{\text{reg}}$$

Detection term

The purpose of the detection term E_{det} is to keep the trajectories $\mathbf{X}_i$ close to the observations $\mathbf{D}_g^t$. It is defined as:

$$E_{\text{det}}(\mathbf{X}) = \sum_{i=1}^{N} \sum_{t=s_i}^{e_i} \left[\lambda - \sum_{g=1}^{D(t)} p_g^t \frac{s^2}{\left\|\mathbf{X}_i^t - \mathbf{D}_g^t\right\|^2 + s^2} \right]$$

where:

- s is a constant referring to the diameter of the NPs,
- λ is a constant,
- p_g^t is the U-Net prediction associated to the detection $\mathbf{D}_g^t$.

Intensity term

The intensity term E_{int} is to provide intensity (or mass) conservation along a trajectory. It is defined as the sum of the relative intensity differences along the trajectory:

$$E_{\mathrm{int}}(\mathbf{X}) = \sum_{i=1}^{N} \sum_{t=s_i}^{e_i-2} \left(\frac{I(\mathbf{X}_i^t) - I(\mathbf{X}_i^{t+1})}{I(\mathbf{X}_i^t) + I(\mathbf{X}_i^{t+1})} \right)^2$$

where $I(\mathbf{X}_i^t)$ is the total intensity of the object located at $\mathbf{X}_i^t$, this intensity being estimated after subtracting the local background around the object.

Dynamic model term

The dynamic model term E_{dyn} tends to minimize the distance between successive detections in agreement with the Brownian motion model. It is defined as:

$$E_{\mathrm{dyn}}(\mathbf{X}) = \sum_{i=1}^{N} \sum_{t=s_i}^{e_i-1} \left\| \mathbf{X}_i^t - \mathbf{X}_i^{t+1} \right\|^2$$

Mutual exclusion term

The mutual exclusion term E_{exc} is intended to apply a penalty to configurations in which two NPs come too close to each other. It is taken as:

$$E_{\mathrm{exc}}(\mathbf{X}) = \sum_{t=1}^{F} \sum_{i,j \neq i}^{N(t)} \frac{s^2}{\left\| \mathbf{X}_i^t - \mathbf{X}_j^t \right\|^2 + s^2}$$

Regularization term

The role of the regularization term E_{reg} is to penalize solutions with a too high number of NPs and solutions with short trajectories. It is defined as:

$$E_{\mathrm{reg}}(\mathbf{X}) = N + \sum_{i=1}^{N} \frac{1}{F(i)}$$

where $F(i)$ is the length of trajectory i defined by $F(i) = e_i - s_i + 1$.

Parameter settings

The value of the constants has been determined using the simulated sequences. In all experiments, they are set to the following values:

- α to δ are set to $\{1, 0.02, .5, 1\}$
- $\lambda = 0.125.$
- $s = 7$

SI-D. Construction and study of simulated dynamic sequences

Table SI-T1 reports the input parameters of the simulated dynamic sequence illustrated in the main text and reported as the supplementary video Video03.avi.

General parameters	Exponent α of the 'STEM-ADF' power-law	1.8
	calibration nm/pixel	0.3906
	Simulated field of view (nm^2)	100x100
Supporting media	Atomic density (number of atoms/nm^3)	0.03
	Atomic number Z	13
	Mean thickness (nm)	16
	Maximal rugosity (nm)	2.5
	Average pore size (nm)	11
	Number of pores	54
Nanoparticle population	Mode of distribution of NPs on the support surfaces	*one side*
	Average targeted NP radius (nm)	3.20
	Size dispersion (nm)	0.36
	Resulting NP size (nm)	3.08
	Atomic number Z	22
	Atomic density (number of atoms/nm^3)	0.04
	Initial number of NPs	55
Trajectories	Mean displacement (nm) from one frame to another	0.4
	Maximal random speed variation (%)	40
	Speed reduction for NPs close to pores (%)	75
	Orientation of starting trajectories	*random*
	Forward diffusion: typical maximal deviation angle	
	(in °) with respect to the current motion	30
	Distance under which NPs may coalesce (nm)	0.4
	Probability of coalescence under previous distance	0.35
	Dissolution of smallest NPs	*no*
	Accounting for Ostwald ripening	*no*
	Probability of nucleation of new NPs	0
	Total number of simulated frames	50

Table SI-T1: Main input parameters of the code simulating the sequence illustrated in the main text. Atomic numbers and densities are adequately chosen for reproducing a similar range of background and NP intensities as compared to experimental STEM images. The ’rugosity’ parameter is the amplitude of wavy and more or less random thickness variations. Pores are generated with a rough truncated cuboid shape. The speed reduction for NPs close to pores intends to mimick an anchorage effect. The forward diffusion angle refers to the maximal angular deviation of a trajectory with respect to its current direction.

Figures SI-D1 and SI-D2 are further qualitative and quantitative illustrations of its analysis, again completed by the supplementary video Video04.avi.

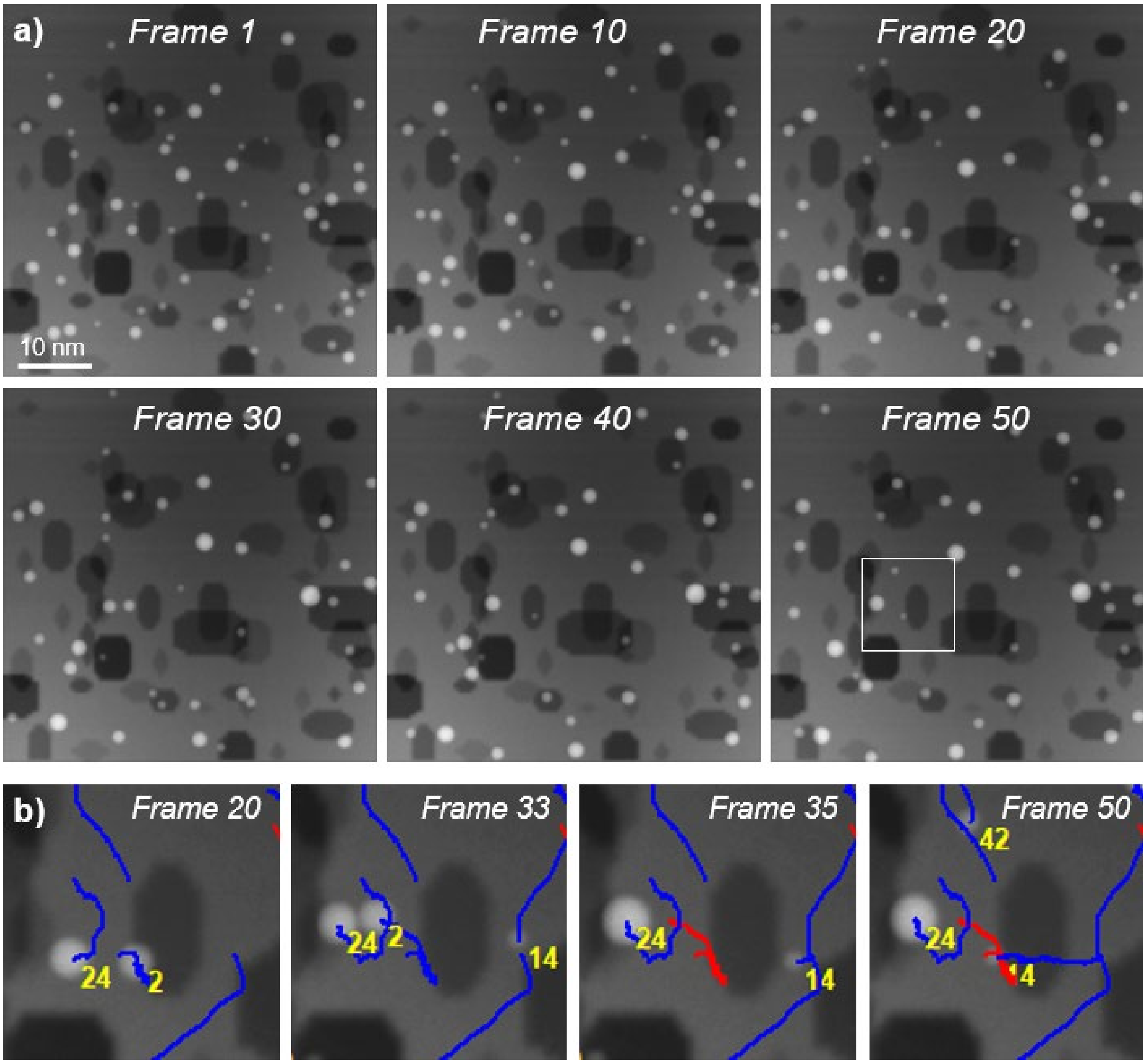

Figure SI-D1: Illustration of a typical simulated sequence of 50 images reported as the video Video03.avi. a): Selection of a few frames showing the general evolution of the NP population (every 10 frames shown). b): Enlarged detail from white frame in a) showing a coalescence event as they can occur with a controlled probability. NP 24 absorbs NP 2; note the slight radius and intensity increase (trajectories in progress are shown in blue, the ended trajectory of NP 2 is shown in red). In this sequence, all NPs are lying on the same surface of the substrate.

Figure SI-D3 illustrates the identification of fusion events with the help of a simulated dynamic sequence of 50 frames summarized in Fig. b-c). In a), a moving NP (labeled 2) approaches another NP (1, almost immobile within a pore of the support). The coalescence has a parameterized probability to occur if the particles become closer to a certain critical distance d_C.

The post mortem analysis identifies such an event through the application of 3 criteria:

(i) A NP must disappear. This implies the detection of frame number f_{dis} where a nanoparticle NP_{dis} has vanished according to the brutal ending of an identified trajectory

(ii) There must be an 'eating' NP. In the frame before that of disappearance, we then have to search for a candidate NP_{eating} closer to NP_{dis} than a parameterized distance d_C

(iii) The fusion must be consistent in terms of conservation of matter: in principle both the volume and the STEM integrated intensity of the resulting NP_{eating} in frame f_{dis} must be the sums of the respective volumes V(NP, f) and intensities I_{STEM}(NP, f) of both NP in the previous frame f_{dis}-1:

$$V(NP_{eating}, f_{dis}) = V(NP_{eating}, f_{dis}-1) + V(NP_{dis}, f_{dis}-1) \quad /1/$$

$$I_{STEM}(NP_{eating}, f_{dis}) = I_{STEM}(NP_{eating}, f_{dis}-1) + I_{STEM}(NP_{dis}, f_{dis}-1) \quad /2/$$

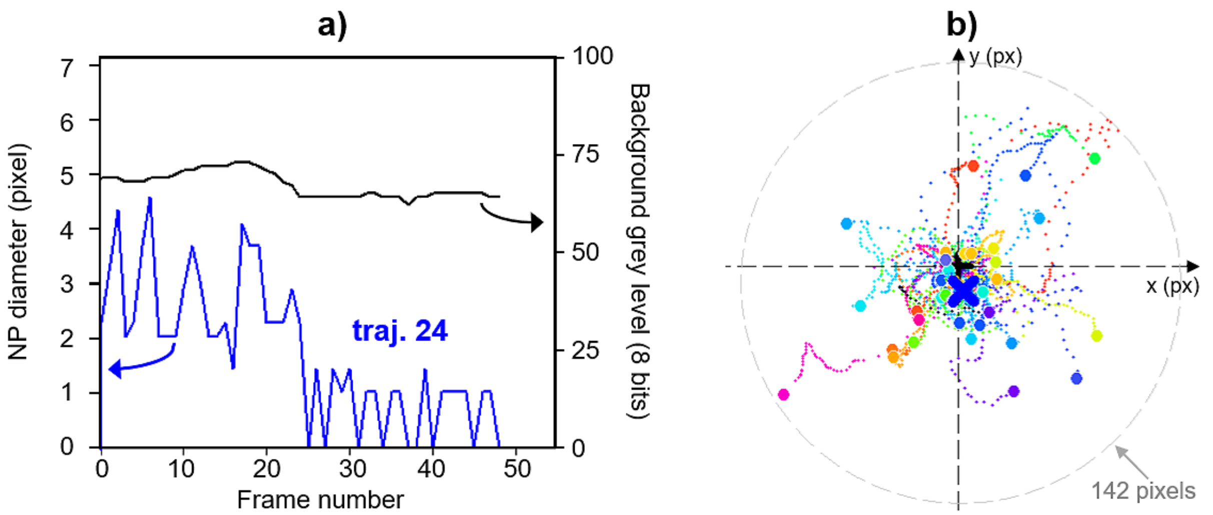

Figure SI-D2: Examples of output from the simulated sequence shown in Fig. SI-D1 (ground truth data). a): Motion of a given NP (#24) through the successive frames; note that its displacements are shorter for darker areas of the support, a correlation introduced on purpose to mimick anchorage of NPs near or within pores. b): Plot of NP (dx,dy) displacements at each frame of the sequence relatively to their initial position in the first frame (small dots; larger dots are for the last frame). The blue cross shows their barycenter at the end of the sequence, very close to the initial barycentre (dark cross at the origin) as expected for a Brownian motion. The grey circle indicates the maximal final displacement.

Relations /1/ and /2/ are most likely rarely exactly verified according to practical reasons. On the one hand, the volume cannot be properly measured from a single 2D image and is only deduced from the estimated Ferret radius of the projections of NPs assuming a purely spherical shape. On the other hand, the evaluation of NP intensities is subjected to errors due to the determination of the local background around each NP and possible intrinsic variations with time in addition to possible variations due to noise changes. Therefore, these relations are replaced by a check that the final measured volume and intensity of the 'eating' NP are sufficiently close to their respective expected values (calculation of the relative error which must be smaller than a parameterized bound).

Figures b-c) show the final 50th frame of the simulated sequence. Fig. b) is the ground truth. All fusion events have properly been identified by our method on the basis of trajectories determined by the NP-Tracker routine (minor differences in the orientation in the green segments are due to a possible delay in detecting the fusion in comparison with the ground truth plots).

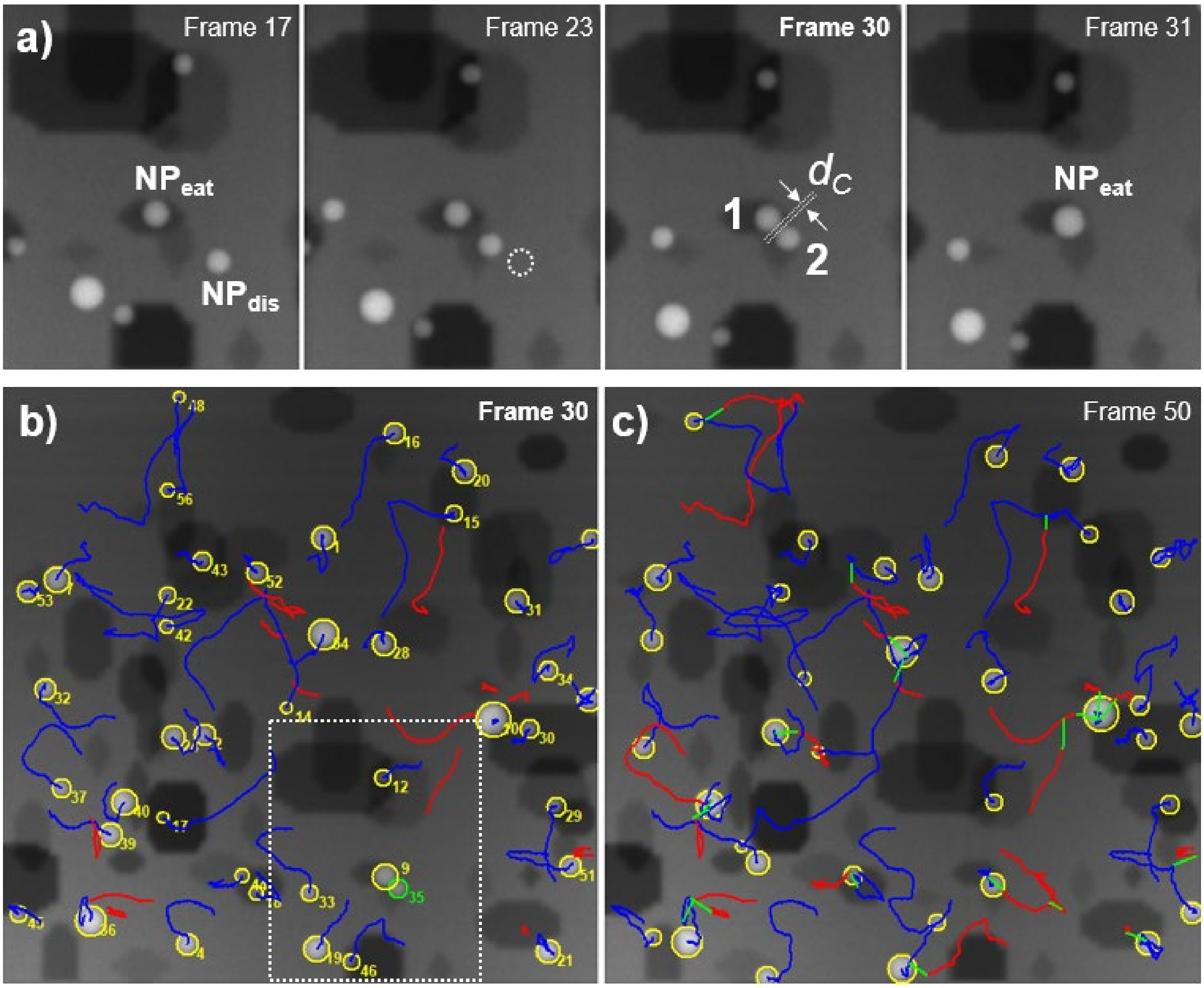

Figure SI-D3: description of the analysis of fusion events. a): enlarged detail of an area of the simulated sequence shown in Figure SI-D1 and reported in b) (dotted rectangle) and c), and followed over a few frames. b-c): Ground truth respectively at frame 30 as enlarged in a) and at the end of the sequence (50th frame). Ongoing trajectories are shown in blue whereas ended ones are displayed in red; green segments indicate the last inter-frames motion of 'eaten' NPs due to fusion events; NP 35 in b) is shown in green just before its absorption by NP 9.

SI-E. Evaluation of simulated dynamic sequences

Table 1 in the main text related to evaluation metrics of the tracking procedure [2] contains the following elements:

- FP (False Positive) and FN (False Negative) respectively represent the number of additional detections (a particle is detected by the algorithm but no particle is present at its location in the Ground Truth) and the number of missed detections (no particle is detected by the algorithm whereas one is present in the Ground Truth).
- IdSwitches (Identity Switches) represent the number of switches in trajectory identifiers i.e. when the identity of two particles are switched between two close trajectories.
- MOTA (Multi-Object Tracking Accuracy) and MOTP (MOT Precision):
  These values vary between 0 and 100 (the higher the better). MOTA corresponds to the normalized sum of the values of the three previous factors quantifying the relevance of the tracking: FP, FN and IdSwitches (for these 3 parameters, the lower value the better).
  MOTP accounts for the spatiotemporal overlap between the Ground Truth and identified tracks over all frames and particles of the series, based on the Mapped Overlap Ratio (see [2]).

SI-F. NP detection efficiency: U-Net vs. local thresholding

We have compared our U-Net based detection to a standard image processing approach. Considering the low contrast and the strong variations in the background, a simple threshold-based method could not work. We thus used a correlation-based approach. The principle is to build a template image composed of a positive disk of radius *r* surrounded by a negative ring of maximum radius 3*r*/2 such as the sum of the values inside the disk is exactly the opposite of the sum of the values inside the ring (see figure SI-F1). The correlation of the input image with this template is equivalent to evaluate the local contrast between the mean gray level of the disk and the mean gray level of the background around this disk. It will then produce local maxima at the location of the particles of radius *r*. The result can be binarized by a simple thresholding to a given contrast *c*. This operation is iterated for a given range of radius to detect all the particles. The final segmentation is obtained as the union of all binary images. Figure SI-F2 illustrates the three steps: correlation, binarization, union of all the binary images obtained for different radius.

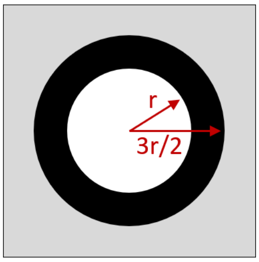

Figure SI-F1: Template composed of a positive disk surrounded by a negative ring.

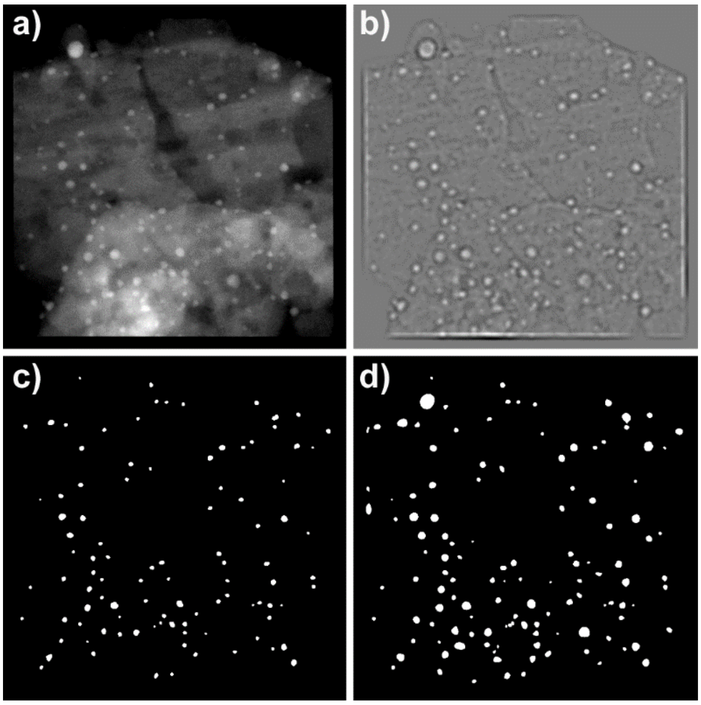

Figure SI-F2: Illustration of the correlation-based segmentation: a) original image; b): typical correlation image; c): binarization of the correlation image; d): union of all binary images.

This routine named 'STRATUS' was programed in MATLAB (R2020b, Mathworks). It was applied to the whole simulated series illustrated in Fig. 6 of the main text, to the first frame of the experimental Pd250 series (for which the Ground Truth was established manually), see figure Si-F3, and to another micrograph taken on a similar catalytic system, i.e. Pt@γ-Al_2O_3 [3], see figure SI-F4. The quantitative comparison between U-Net and the 'STRATUS' code is summarized in Table SI-T2. Note that in the case of the Pd250 series (Fig. SI-F3e) the parametrization of the correlation-based approach was very delicate (the optimal results were expected to exhibit similar but smallest numbers of FN and FP events).

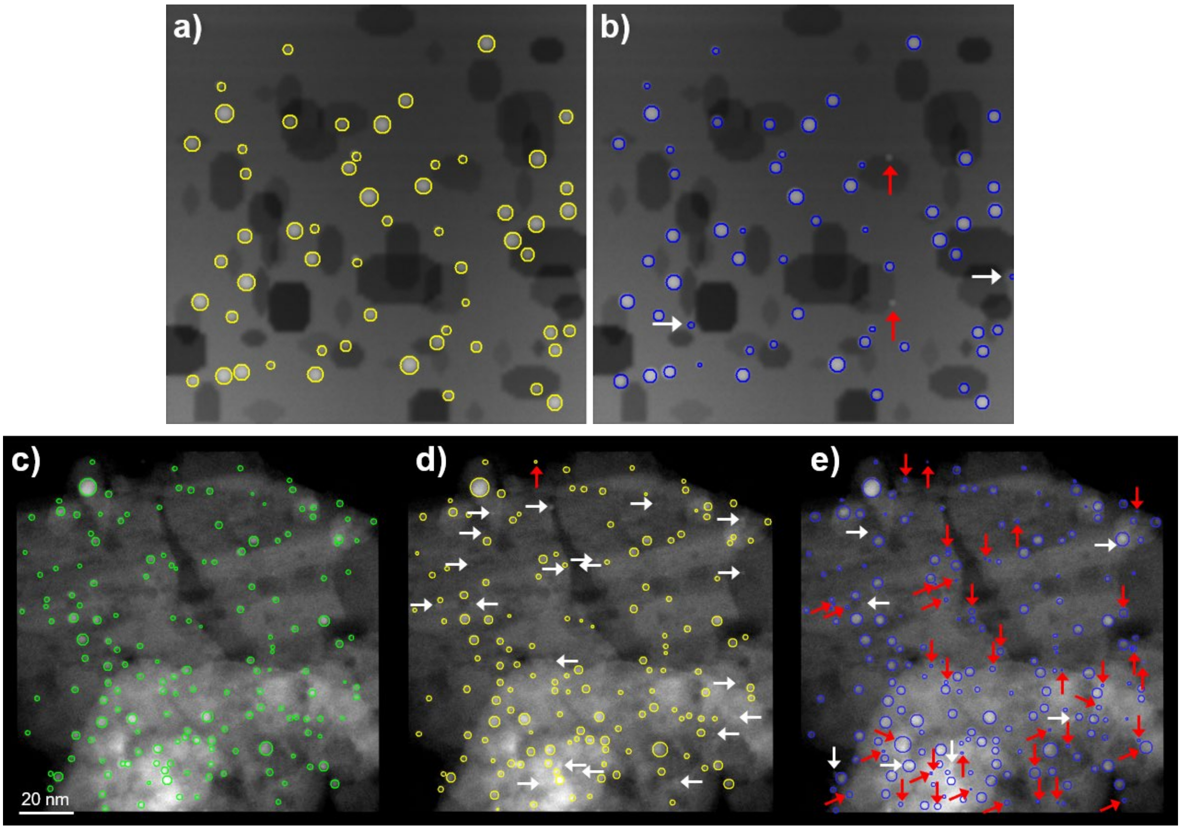

Figure SI-F3: Illustration of the NP detection efficiency for the U-Net and correlation-based approaches.

a-b): Treatment of the first frame of the simulated series shown in Fig. 6 of the main text: a) U-Net results strictly corresponding to the Ground Truth; b): correlation-based results; FN and FP events are marked with red vertical and white horizontal arrows. Results for the whole sequence are given in Table SI-T2.

c-e): Treatment of the first frame of the experimental Pd250 series shown in Fig. 8 of the main text. c): Manual Ground Truth; d): U-Net results; e): correlation-based results. In d) and e) FN and FP events are marked with red vertical or inclined and white horizontal arrows respectively).

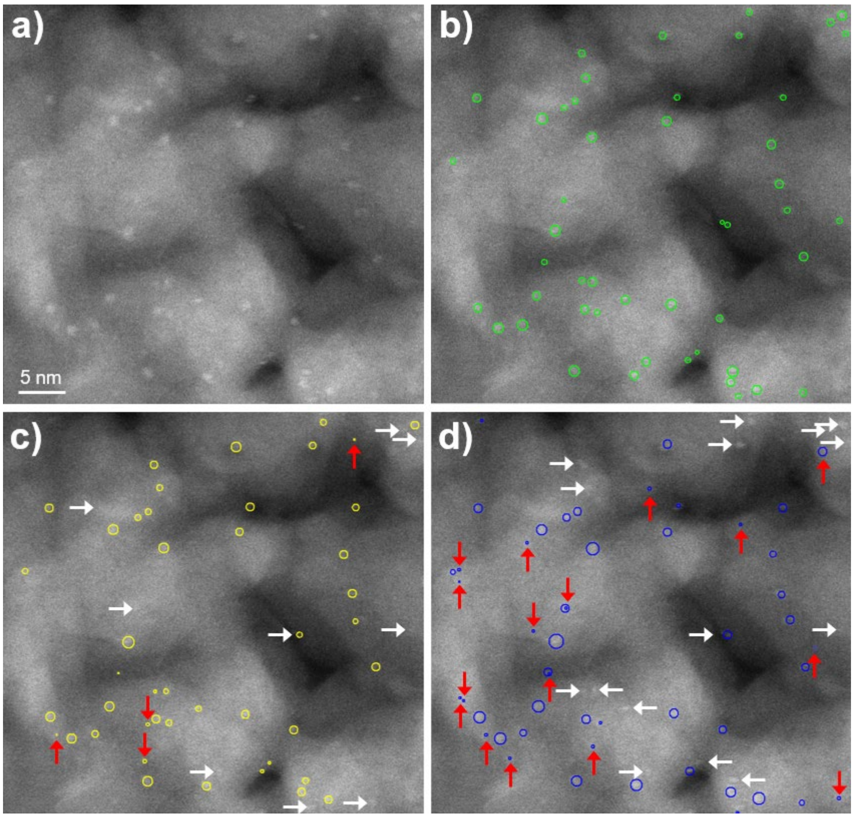

Figure SI-F4: same as fig. SI-F3 for the Pt@g-Al_2O_3 system.

a): Raw STEM image showing metallic clusters in a Pt@g-Al_2O_3 system observed at 50°C under 5 mbar of hydrogen (adapted from [3]). b): Manual Ground Truth; c): U-Net results; d): correlation-based results. In c) and d) FN and FP events are marked with red vertical inclined and white horizontal arrows respectively).

		Ground Truth	U-Net	STRATUS code
**simulated sequence (50 images)**	**FP**		0	74
	**FN**		9	29
	**Total number of NPs**	2222	2213	2267
	**Errors %**		0.4%	4.5%
**Pd250, frame 1**	**FP**		1	40
	**FN**		20	7
	**Total number of NPs**	162	143	195
	**Errors %**		14.7%	24.1%
**Pt@ γ-Al_2O_3**	**FP**		4	16
	**FN**		9	15
	**Total number of NPs**	48	43	49
	**Errors %**		30.2%	63.3%

Table SI-T2: Quantitative comparison of the U-Net approach and correlation-based MATLAB code for the detection of particles in the examples shown in Figure SI-F3.

SI-G. Estimation of the SNR for particle detection

The detection of a given NP of index *k* depends on the ratio between the intrinsic intensity of this particle and the standard deviation σ_k of the noise of the background taken locally around this particle. To give a global value of the signal-to-noise ratio (SNR) over a whole image, we will use a constant value σ averaged over all σ_k values and thus consider that the noise is stationary. The corresponding signal to noise ratio (SNR) SNR_k is then defined as:

$$\mathrm{SNR}_k = 20\log_{10}(A_k/\sigma)$$

Figure SI-G1 illustrates the analysis. The average intensity A_k of each particle is calculated by dividing the integrated intensity $I_{STEM,k}$ by the projected NP surface, all values being determined during the U-Net / Np-Tracker treatment of the experimental Pd250 series (see the 'Treacy-Rice' plot in figure 8 in the main text). It can be rendered visually: Fig. SI-G1 b) is an impainted vision of the initial micrograph in a) where each pixel inside a NP detected by the treatment is replaced by the local mean background measured around it. Then, Fig. c) is the difference a)-b). Note that small NPs, which appear with a reasonably good visibility in a), have indeed a low intensity hardly visible on a null background. Figure SI-G1 e) shows that most of the particles have a relatively high SNR (the median is 8.1 dB), but a significant number of particles have a very low SNR (the first decile is at 0.6 dB which is a low value) making them hardly detectable by any local threshold-based image processing.

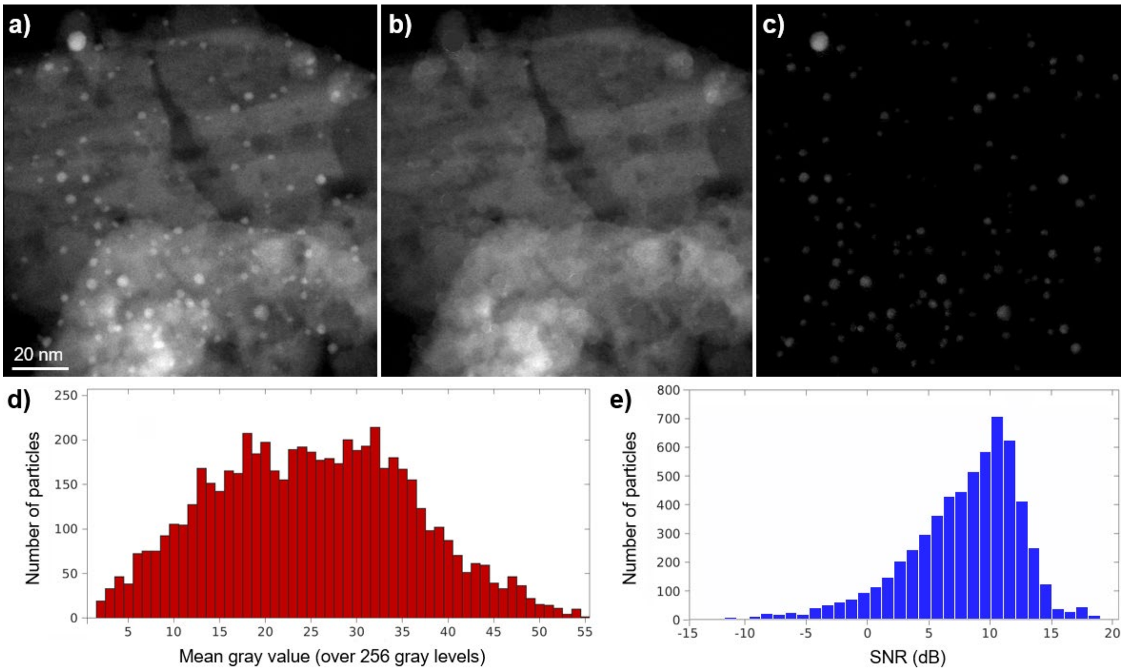

Figure SI-G1: Analysis of the SNR of the detected NP in the Pd250 series.

a-c): illustration of the treatment for the first frame of the series. a): raw STEM micrograph. b): Impainted particle-free image. Given the segmentation mask of each particle (output of U-Net detection step) in the original image, the intensity of pixels in each NP $I_k(i,j)$ s replaced by the mean gray level in the background surrounding the mask and denoted B_k. c): Intensities of the NPs on a null background; the pixel intensities are taken as the difference $I_k(i,j)$-B_k.

d): Distribution of averaged intensities A_k of all detected NPs over the 69 frames of the Pd250 sequence *(*A_k is the average of $I_k(i,j)$-B_k over all pixels of NP *k).*

e): Distribution of particle SNR over all NPs of the Pd250 image sequence.

References

[1] A. Milan, S. Roth, and K. Schindler, "Continuous energy minimization for multitarget tracking", *IEEE Transactions on Pattern Analysis and Machine Intelligence*, 36, 1, 58-72, https://doi.org/10.1109/TPAMI.2013.103, 2014.

[2] R. Kasturi *et al.*, "Framework for Performance Evaluation of Face, Text, and Vehicle Detection and Tracking in Video: Data, Metrics, and Protocol", *IEEE Transactions on Pattern Analysis and Machine Intelligence*, 31, 2, 319-336. https://doi.org/10.1109/TPAMI.2008.57 (2009).

[3] C. Dessal *et al.*, "Atmosphere-dependent stability and mobility of catalytic Pt single atoms and clusters on γ-Al_2O_3", *Nanoscale*, 11, 14, 6897-6904. https://doi.org/10.1039/C9NR01641D (2019).
